# Effects of Withdrawal from Cocaine Self-Administration on Rat Orbitofrontal Cortex Parvalbumin Neurons Expressing *Cre recombinase*: Sex-Dependent Changes in Neuronal Function and Unaltered Serotonin Signaling

**DOI:** 10.1523/ENEURO.0017-21.2021

**Published:** 2021-07-08

**Authors:** Andrew M. Wright, Agustin Zapata, Alexander F. Hoffman, Julie C. Necarsulmer, Lamarque M. Coke, Reinis Svarcbahs, Christopher T. Richie, James Pickel, Bruce T. Hope, Brandon K. Harvey, Carl R. Lupica

**Affiliations:** 1 Electrophysiology Research Section, Cellular Neurobiology Branch with: Cellular and Neurocomputational Systems Branch; 2 Molecular Mechanisms of Cellular Stress and Inflammation Section; 3 Optogenetics and Transgenic Technology Core; 4 Neuronal Ensembles in Drug Addiction Section Integrative Neuroscience Branch, National Institute on Drug Abuse, Intramural Research Program, National Institutes of Health, Baltimore, MD 21224; 5 Transgenic Technology Core, Intramural Research Program, National Institute of Mental Health, Bethesda, MD 20892

**Keywords:** addiction, electrophysiology, orbitofrontal cortex, parvalbumin, self-administration, serotonin

## Abstract

The orbitofrontal cortex (OFC) is a brain region involved in higher-order decision-making. Rodent studies show that cocaine self-administration (CSA) reduces OFC contribution to goal-directed behavior and behavioral strategies to avoid drug intake. This change in OFC function persists for many weeks after cocaine withdrawal, suggesting involvement in the process of addiction. The mechanisms underlying impaired OFC function by cocaine are not well-understood. However, studies implicate altered OFC serotonin (5-HT) function in disrupted cognitive processes during addiction and other psychiatric disorders. Thus, it is hypothesized that cocaine impairment of OFC function involves changes in 5-HT signaling, and previous work shows that 5-HT_1A_ and 5-HT_2A_ receptor-mediated effects on OFC pyramidal neurons (PyNs) are impaired weeks after cocaine withdrawal. However, 5-HT effects on other contributors to OFC circuit function have not been fully investigated, including the parvalbumin-containing, fast-spiking interneurons (OFC^PV^), whose function is essential to normal OFC-mediated behavior. Here, 5-HT function in naive rats and those withdrawn from CSA were evaluated using a novel rat transgenic line in which the rat parvalbumin promoter drives Cre-recombinase expression to permit identification of OFC^PV^ cells by fluorescent reporter protein expression. We find that whereas CSA altered basal synaptic and membrane properties of the OFC^PV^ neurons in a sex-dependent manner, the effects of 5-HT on these cells were unchanged by CSA. These data suggest that the behavioral effects of dysregulated OFC 5-HT function caused by cocaine experience are primarily mediated by changes in 5-HT signaling at PyNs, and not at OFC^PV^ neurons.

## Significance Statement

Cocaine addiction involves the inability to change behavior having negative consequences and the reluctance to adopt beneficial behaviors. The orbitofrontal cortex (OFC) is a brain region involved in this behavioral flexibility, and OFC function is impaired after cocaine use. Moreover, signaling by the neurotransmitter serotonin (5-HT) is impaired in OFC pyramidal neurons (PyNs) after cocaine. However, whether other types of OFC neurons are affected by cocaine is unknown, and we asked whether changes occur in another class of OFC cells known as parvalbumin interneurons. We report that cocaine changed the activity of parvalbumin interneurons in a sex-dependent manner but did not alter 5-HT effects. This suggests that the effects of cocaine on 5-HT function in OFC involves PyNs and not parvalbumin interneurons.

## Introduction

The orbitofrontal cortex (OFC), a prefrontal cortical (PFC) area, is a brain region involved in executive cognitive function in domains such as associative learning, reversal learning, outcome prediction, and estimation of reward value (Jones and Mishkin, 1972; Schoenbaum et al., 1998, 2016; Gallagher et al., 1999; Tremblay and Schultz, 1999; Izquierdo and Murray, 2004; Hardung et al., 2017). A hallmark of OFC function is its contribution to flexible behavior as defined by the ability to shift behavioral strategies in the face of changing contingencies and expected outcomes (Ostlund and Balleine, 2007; Schoenbaum and Esber, 2010; Gremel and Costa, 2013; Izquierdo, 2017).

The OFC is innervated by all major monoaminergic nuclei (Chandler et al., 2013) and sends projections to most of these areas (Dalley et al., 2004; Hoover and Vertes, 2011). Of relevance is the dense innervation the OFC receives from the 5-HT neurons of the dorsal raphe nucleus (Azmitia and Segal, 1978; Hornung et al., 1990; Wilson and Molliver, 1991a,b; Gonçalves et al., 2009; Vázquez-Borsetti et al., 2009; Roberts, 2011; Chandler et al., 2013). A growing body of evidence shows that 5-HT is critical for OFC-dependent behavior and that dysregulation of 5-HT function is implicated in psychiatric disorders where changes in OFC-dependent executive function are observed (Roberts, 2011). Additionally, pharmacologically targeting the 5-HT system is associated with positive treatment outcomes and increased OFC metabolism in psychiatric disorders (Saxena et al., 1999; New et al., 2004). In animal studies, OFC-dependent behavioral tasks that require shifts in behavioral strategies to obtain reward are impaired by the depletion of 5-HT (Clarke et al., 2004; Roberts, 2011; Izquierdo et al., 2012; West et al., 2013). Moreover, selective ligand activation of 5-HT receptor subtypes within the OFC alters reversal learning, a form of behavioral flexibility (Boulougouris and Robbins, 2010; Furr et al., 2012), and other OFC-dependent behaviors such as reinforcer devaluation and response inhibition are blocked by lesions of OFC 5-HT axons (West et al., 2013; Man et al., 2010).

The inability to change behavior in the absence of reward or in the presence of aversive consequences is endemic to cocaine use disorder (CUD) in humans, and cocaine exposure is associated with the dysregulation of OFC function in humans and in animal models (Volkow and Fowler, 2000; Bechara, 2005; Schoenbaum and Shaham, 2008; Lucantonio et al., 2012, 2014; Simmler et al., 2017). Thus, CUD is associated with a reduction in OFC metabolic activity that persists after cocaine withdrawal (Volkow et al., 1993; Volkow and Fowler, 2000), and enduring impairments in performance of behavioral tasks relying on intact OFC function are observed in animals following cocaine withdrawal. This includes reductions in the ability to change strategies to obtain reward (e.g., reversal learning; Jentsch et al., 2002; Schoenbaum et al., 2004; Calu et al., 2007), and in performance of non-operant behaviors such as reinforcer devaluation (Schoenbaum and Setlow, 2005) and overexpectation learning (Takahashi et al., 2013; Lucantonio et al., 2014). These findings point toward long-lasting changes in OFC function contributing to disordered behavior following cocaine exposure in humans and in animals (Lucantonio et al., 2014). However, the neural mechanisms through which this occurs are poorly understood.

The changes in OFC-dependent behavior observed after cocaine experience are hypothesized to result from altered serotonergic control of OFC neuron activity as large reductions in 5-HT-mediated electrophysiological effects and altered expression of 5-HT_2A_ receptor transcripts are observed in OFC pyramidal neurons (PyNs) weeks after withdrawal from cocaine self-administration (CSA) or yoked cocaine administration (Wright et al., 2017). However, the output of OFC, like other cortical circuits, relies on the interaction of glutamatergic PyNs (principal cells) with less-numerous local circuit GABAergic interneurons. One class of these interneurons, known as fast-spiking parvalbumin neurons (OFC^PV^), regulates PyN excitability via inhibitory synapses and plays an essential role in the generation of γ oscillations that promote frontocortical synchrony necessary for OFC-dependent behavior (Bissonette et al., 2015). As cortical parvalbumin neurons also express 5-HT receptors (Santana et al., 2004; Athilingam et al., 2017), and promote cortical synchrony (Puig et al., 2010), we hypothesize that withdrawal from CSA will also alter 5-HT responses in these cells. To evaluate this, a novel transgenic rat (Pvalb-iCre) was developed in which Cre-recombinase expression was driven by the parvalbumin promoter, and a Cre-dependent viral targeting strategy was used to examine 5-HT function in these cells following withdrawal from CSA. We report that although sex-dependent differences in OFC^PV^ neuron properties were observed after CSA, and effects of 5-HT on these cells differed between male and female cocaine-naive rats, no changes in response to 5-HT were observed after cocaine withdrawal. Therefore, we conclude that the enduring effects of cocaine on OFC 5-HT function and behavior likely occur primarily through actions at PyNs and not OFC^PV^ cells.

## Materials and Methods

### Generation of a bacterial artificial chromosome (BAC) for Pvalb-iCre

**Figure 1.**
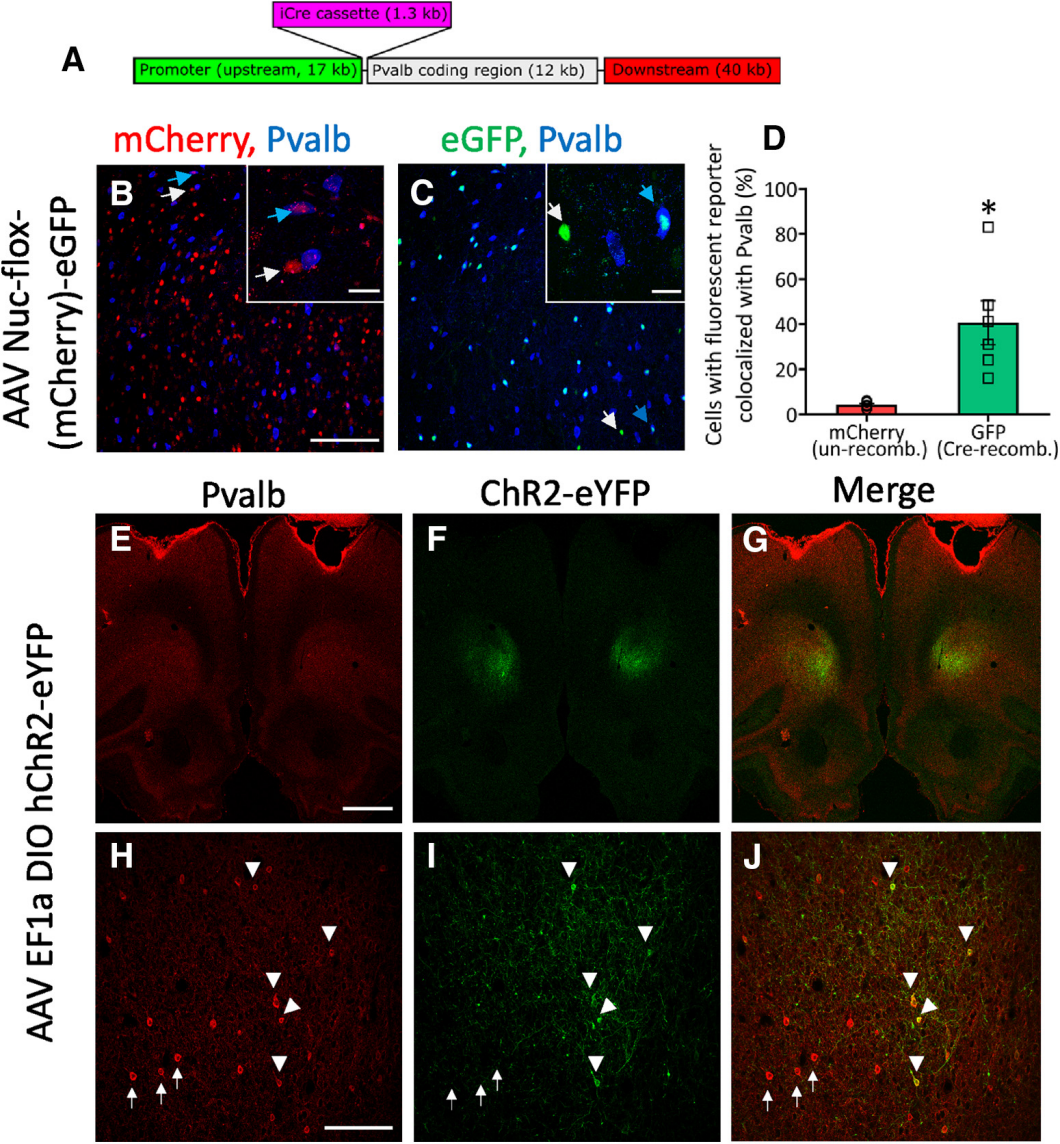
Generation of Pvalb-Cre rats. ***A***, Schematic of the Pvalb-iCre transgene produced by recombineering BAC CH230-499N20 which contains 17 kb of endogenous sequence upstream of the Pvalb start codon. Pvalb-iCre rats were injected bilaterally with AAV Nuc-flox-(mCherry)-eGFP into the OFC and brains processed for fluorescent imaging four weeks later. ***B***, Unrecombined AAV genomes express mCherry in the OFC (red) and have minimal colocalization with Pvalb-positive cells (blue). Inset shows high magnification of a mCherry+, Pvalb+ cell (blue arrow), and a mCherry+, Pvalb– cell (white arrow). ***C***, Cre-recombination leads to GFP-positive cells (Cre recombined; green) which showed colocalization with Pvalb (blue). Inset is high magnification example of a GFP+, Pvalb+ cell (blue arrow) and a GFP+, Pvalb– cell (white arrow). ***D***, Quantitation of colocalization of mCherry (unrecombined) and GFP (Cre-recombined) with Pvalb-positive cells. *p = 0.038, paired t-test. ***E–J***, Rats were injected with AAV EF1a DIO hChR2-eYFP and immunostained for parvalbumin 20 d later. At low magnifications, diffuse Pvalb-immunoreactivity (***E***) and focal ChR2-EYFP (***F***) expression in the OFC. At higher magnification, distinct Pvalb-immunopositive cells are present in OFC (***H***) and a mesh-like pattern of fluorescence from ChR2-EYFP expressed throughout cell bodies and processes in the OFC (***I***). In cells clearly exhibiting somal expression of ChR2-EYFP (white triangle), there is corresponding expression of Pvalb (***J*)**. Pvalb-positive cells that do not colocalize with ChR2-EYFP are indicated white arrow). Scale bars: 50 μm (***B***, ***C***, ***H***, ***J***), 20 μm (inset), and 1000 μm (***E–G***).

A BAC carrying a 70-kb fragment of rat genomic DNA containing the *Pvalb* gene locus was obtained from the Children’s Hospital Oakland Research Institute (CH230-499N20). The BAC was transformed into the recombineering bacterial strain (GS1783) using electroporation and edited using “en passant mutagenesis” to replace the start codon of the *Pvalb* gene (Tischer et al., 2010; Shimshek et al., 2002). Specifically, a cassette containing an I-SceI restriction site and a KanS selection marker was amplified from pEP-KanS (a gift from Nikolaus Osterrieder; Addgene plasmid #41017; http://n2t.net/addgene:41017; RRID:Addgene_41017) and inserted into a plasmid containing the coding region for iCre (pBS mFos TetO iCre, pOTTC161). A 50-bp portion of the iCre coding region was duplicated on both sides of the insertion to facilitate intramolecular recombination and marker removal in subsequent steps. The final plasmid, pBS mFos TetO iCre(KanS) (pOTTC4094) was used as a template to PCR amplify the entire iCre-(I-SceI-KanS) coding region with primers appended with 5′ linkers corresponding to 50 bp of homology with the sequences adjacent to the *Pvalb* start codon. This PCR product was electroporated into the GS1783 cells containing the Pvalb BAC after heat shock, and the transformants were selected on LB agar plates containing chloramphenicol and kanamycin. Successful transformants were screened by PCR across the 5′ and 3′ junctions and sequence verified for error-free insertion. The kanamycin resistance marker was removed, and the iCre coding region restored by the simultaneous treatment with arabinose and heat shock. Cultures were diluted and streaked onto plates containing LB agar plates containing chloramphenicol. Successful recombinants were screened by PCR and verified by sequencing and pulse-field agarose gel electrophoresis. The final Pvalb-iCre BAC (pOTTC465) was prepped using Macherey–Nagel NucleoBond Xtra BAC columns and then used to create the transgenic rat line. See schematic of final transgene (Fig. 1, transgene and characterization).

### Transgenic rat husbandry

All animal procedures were performed in accordance with National Institutes of Health *Animal Care Guidelines* and approved by local animal care and use committees. Procedures were initially based on those for optimizing the production of transgenic rats (Filipiak and Saunders, 2006) with modifications described here. Female Long–Evans rats purchased from Charles River Laboratories and maintained in a 6 A.M. to 6 P.M. light cycle with food and water ad lib were superovulated and mated as described (Filipiak and Saunders, 2006). Briefly, the ovulation of females, four to five weeks of age, was synchronized using 40-μg LHRH (Luteinizing Hormone Releasing Hormone, Sigma Chemicals) injected intraperitoneally on day 1. Superovulation was achieved with 20 IU pregnant mare serum gonadotropin (PMSG; National Hormone and Peptide Program) 48 h later at 10 A.M. Another 48 h later, 20 IU human chorionic gonadotropin (hCG; Novarel, Ferring) was injected intraperitoneally. Directly after these injections the females were added to cages with singly housed Long–Evans stud males. The next morning at 9 A.M. copulation plugs were identified, and fertilized oocytes collected from the females in M2 medium and then treated with M2/hyaluronidase to remove cumulus cells. They were then transferred to KSOM medium (all medium from Sigma) and incubated at 37° with 5% CO_2_ in a humidified incubator. By 10 A.M. when pronuclear membranes were visible, they were injected with a solution (2–3 ng/μl) of the BAC DNA described above. Later in the day zygotes were surgically transferred to the oviducts of female rats. These recipients and vasectomized males were housed in a 3 A.M. to 3 P.M. light cycle. The females’ ovulation synchronized as described above with 80 μg LHRH on day 1 at 10 A.M. and mated with vasectomized SD males on day 5. These recipient females were monitored daily after surgery. Pups were weaned at p21 and ear punches were used to identify and provide biopsies for genotyping as described below. Animals found to be positive for the transgene were then identified with ear tags. Animals were bred as Cre-positive carriers by wild-type Long–Evans from Charles River Laboratories.

### Genotyping and copy number analysis

Genomic DNA was isolated from tissue biopsies or primary cells using a Macherey–Nagel Tissue Spin kit. Two genotyping protocols were used to identify animals carrying the Pvalb-iCre transgene. The 5′ junction was amplified with Pvalb F16505 (5′-CAGAACTCTCCAGTGTCTGCTGG-3′) and iCre R173 (5′-CTTCCAGGTGTGTTCAGAGAAG-3′) to produce a 476-bp amplicon. The 3′ junction was amplified with iCre F738 (5′-GTTCTGCCGGGTCAGAAAGAATGGT-3′) and Pvalb R16998 (5′-CCAACCCCGAGGATAAGGGAATG-3′) to produce a 713-bp amplicon. All PCRs used OneTaq (New England Biolabs), and 40 cycles of 94°C for 30 s and 68°C for 1 min. Amplification products were analyzed by agarose gel electrophoresis. For copy number determination, the genomic DNA was isolated as described above served as template for droplet digital PCR using ddPCR Supermix for Probes (No dUTP; Bio-Rad #1863024) and FAM-labeled or HEX-labeled probe assays for reference gene Ggt1 (Witten et al., 2011) and iCre (Table 1). Droplets were generated and analyzed using a QX200 AutoDG Droplet Digital PCR System (Bio-Rad). Amplification reactions were run in a Bio-Rad T100 thermocycler.

**Table 1:** Primers and probes for droplet digital PCR for quantification of copy number

Primer name	Primer Seq	Target gene
Ggt1 probe (HEX, IBFQ)	CCGAGAAGCAGCCACAGCCATACCT	Ggt1
Ggt1pF	CCACCCCTTCCCTACTCCTAC	Ggt1
Ggt1pR	GGCCACAGAGCTGGTTGTC	Ggt1
iCrepF	ATGGTGCCCAAGAAGAAGAG	iCre
iCre probe (FAM, IBFQ)	AAGTCTCCAACCTGCTGACTGTGC	iCre
iCrepR	CTTCCTGACTTCATCAGAGGTG	iCre

### Histologic analysis of brains

#### Cre-dependent AAV reporters for initial phenotyping of putative lines

Parvalbumin-iCre rats used in the present experiments were injected with Cre-dependent fluorescent reporters to identify Cre recombination present in the OFC and other brain regions. For example, an AAV vector expressing a nuclear-localized, color-changing reporter, AAV Nuc-flox-(mCherry)-eGFP (Bäck et al., 2019) was injected into OFC and four weeks later the brains were processed for fluorescent protein expression. To fix brain tissue, animals were anesthetized using isoflurane, then transcardially perfused with heparinized PBS, followed by 4% paraformaldehyde (PFA), and then postfixed for 2 h in 4% PFA. Extracted brains were immersed in succeeding gradients of 18% to 30% sucrose for cryopreservation before being flash frozen. Brains were cryosectioned at 30 μm and washed for 30 min in PBS, followed by a 1-h incubation in a 4% goat serum 0.3% Triton X-100 blocking solution. After 30 min of washing, sections were incubated in primary antibody in blocking solution (mouse anti-parvalbumin, 1:1000, Sigma, P3088) overnight, shaking at 4°C. Sections were then washed in 3× in PBS, incubated for 1 h in a secondary antibody blocking solution (goat anti-mouse, 1:500, Alexa Fluor 680, Invitrogen, A21058) and washed again in PBS. Sections were then stained with DAPI and imaged at 20× magnification using laser confocal microscopy (Nikon Eclipse E800 microscope body, Nikon C2 Confocal microscope system, NIS-Elements imaging software version 4.40; Nikon Instruments Inc.). Cell counting and quantification of parvalbumin and red-to-green signal colocalization was performed in six rats (three males, three females). Six representative brain sections from each animal were used for quantification (ImageJ 1.53C). For single channel quantification, automatic cell counting of particles larger than 3 μm in diameter was applied after adjusting for the background. For colocalization, manual counts were performed. Data are presented as mean % (±SEM) of red or green cells colocalized with parvalbumin staining.

#### Channelrhodopsin2 (hChR2) and endogenous Pvalb expression

For examining colocalization of hChR2 expression with OFC Pval+ neurons, parvalbumin-iCre rats Line 2 were euthanized 20 d following bilateral injection of AAV5-EF1α-DIO-hChR2(H134R)-EYFP (Addgene) into the OFC for immunohistochemical analysis. Brains were collected and sectioned as described above. For immunolabelling, 30-μm cryosections were washed for 30 min in 1 × PBS, followed by a 1-h incubation in a 4% goat serum 0.3% Triton X-100 blocking solution. After 30 min of washing, sections were incubated in primary antibody in blocking solution (mouse anti-parvalbumin, 1:1000, Sigma, P3088) overnight, shaking at 4°C. Sections were then washed in 1 × PBS, incubated for 1 h in a secondary antibody blocking solution (goat anti-mouse, 1:500, Alexa Fluor 568, Invitrogen, A11004), and washed again in 1 × PBS. Sections were then stained with DAPI and imaged using confocal microscopy as described above.

### Subjects

All experimental procedures were approved by the local animal care and use committee and housed in facilities certified by the American Association for the Accreditation of Laboratory Animal Care (AAALAC). Male and Female Pvalb-iCre rats (generated as described above), 140–300 d of age at the time of electrophysiological recording, were housed two to three per cage for at least one week before experimental procedures. They were maintained in a temperature-controlled and humidity-controlled environment under a reverse 12/12 h light/dark cycle (lights on at 9 P.M., lights off at 9 A.M.) with food and water available *ad libitum*. All behavioral experiments were run during the dark phase of the light cycle and begun each day between 10 and 12 A.M. For animals in CSA groups, one week following intravenous catheterization surgery food was restricted to 15 g of standard rodent diet per day and made available after the daily CSA sessions. Animals were food restricted until the end of self-administration training, after which *ad libitum* feeding resumed. All behavioral experiments were conducted during the dark cycle.

### Surgery

#### Intracranial surgery

Rats were anesthetized with isoflurane (3% concentration at 1.5 l of 100% O_2_/min for induction, 2.5% at 1.5 l/min for maintenance) which was applied with a Parkland Scientific V300PK Anesthesia Machine and maintained using a Somni EPS-3 Exposure Prevention System (Somni Scientific). Small holes were drilled into the skull and a stainless-steel cannula was positioned using the coordinates; +4.0 mm anterior to bregma, 2.0 mm lateral to the midline skull suture, and ventral −3.3 mm from the brain surface using a stereotaxic apparatus (Stoelting). Bilateral Injections of the ChR2 viral construct (AAV5-EF1α-DIO-hChR2(H134R)-EYFP, titer 1.8 × 10^13^, Addgene), at a volume of 700 nl, were performed using a Hamilton 1-μl syringe at a rate of 150 nl/min. Following surgery, rats were given one injection of 4-hydroxy-2-methyl- N-(5-methyl-2-thiazolyl)-2H-1,2-benzothiazine-3-216 carboxamide-1,1-dioxide (Meloxicam, 5mg/mL, Boehringer/Ingelheim, Ingelheim, FRG) for post-surgical analgesia, and then single housed with *ad libitum* access to food. The animals were then returned to their home cages and at least two to three weeks were permitted for full expression of the viral construct.

#### Intravenous self-administration (IVSA) surgery

Rats were anesthetized with equithesin (1% pentobarbital, 2% magnesium sulfate, 4% chloral hydrate, 42% propylene glycol, 11% ethanol, 3 ml/kg, i.p.), and a SILASTIC catheter (inner diameter, 0.020 inch; outer diameter, 0.037 inch; Dow Corning) was advanced 3.5 cm into the right jugular vein and secured with silk suture (5–0). Anesthetic depth was assessed continuously throughout the procedure. The catheter terminated in a modified L-shaped 22-G guide cannula (Plastics One) mounted on the skull with cranioplastic cement and secured with three stainless steel screws. Animals were given one injection of meloxicam; for postsurgical analgesia, and were single housed for the remainder of the experiment. Catheters were flushed daily with 0.1 ml of heparinized saline, to maintain patency, and with cefazolin to forestall infection, until the end of operant training. Animals recovered for at least one week after the surgery, and before food restriction commenced. Postsurgery, all animals were monitored for 3 d for signs of adynamic ileus; no such signs were observed in any subjects. Operant training started 2–3 d after the beginning of food restriction.

### CSA training

Self-administration training took place in standard rat operant chambers equipped with two retractable levers (Med Associates). Each chamber was located in a sound attenuating enclosure, equipped with a ventilation fan that provided background noise. Catheters were connected to a length of metal coil-protected plastic tubing (Plastics One) via a liquid swivel (Instech) to a syringe mounted in an infusion pump (Med Associates) located outside the sound attenuating enclosure. Animals were trained to press one lever (active lever) for intravenous cocaine infusions under a fixed ratio 1 (FR1) schedule. At the beginning of the session, the two levers were inserted into the chamber and a house light located on the wall opposite the levers was illuminated. Each lever press resulted in infusion of 0.75 mg/kg cocaine (2.25 mg/ml in sterile saline) accompanied by retraction of both levers, extinction of the house light and illumination of a cue light above the active lever for 30 s. Responses on the inactive lever had no consequence. To facilitate acquisition of the response, animals were allowed to self-administer during 6-h sessions until they received 40 infusions within a session. Most of the animals acquired self-administration within one to two sessions. After that, sessions were limited to 3 h or 75 infusions, whichever occurred first. CSA under the FR1 schedule continued for a total of 12 daily sessions. Following behavioral training, rats in the CSA group remained single housed in their home cages, with *ad libitum* food and water available, until electrophysiology experiments.

### Brain slice preparation

Animals were anesthetized with isoflurane and decapitated using a guillotine. The brains were then extracted and transferred to ice-cold NMDG cutting solution (93 mm NMDG, 2.5 mm KCl, 1.2 mm NaH_2_PO_4_, 30 mm NaHCO_3_, 20 mm HEPES, 25 mm glucose, 5 mm ascorbic acid, 3 mm sodium pyruvate, 10 mm MgCl_2_, and 0.5 mm CaCl_2_). The tissue was then cut perpendicular to its longitudinal axis at ∼1.4 mm posterior to bregma using a razor blade, and then the sectioned surface was glued to the stage of a vibrating tissue slicer (Leica VT1200S, Leica Biosystems). Six to seven coronal sections (250 μm) were collected from each brain beginning at the appearance of the dorsolateral orbital cortex and the rhinal fissure (4.7 mm anterior to bregma) and ending at ∼3.2 mm anterior to bregma. For each animal, recordings were restricted to brain slices corresponding to these landmarks. The slices were then hemisected and transferred to heated (34°C) NMDG for 5 min. The brain slices were then transferred to an oxygenated (95% O_2_/5% CO_2_) holding chamber filled with HEPES-containing artificial CSF (aCSF; 109 mm NaCl, 4.5 mm KCl, 1.2 mm NaH_2_PO_4_, 35 mm NaHCO_3_, 20 mm HEPES, 11 mm glucose, 0.4 mm ascorbic acid, 1 mm MgCl_2_, and 2.5 mm CaCl_2_) at room temperature (23°C) for at least 30 min, and up to 7 h.

### *In vitro* electrophysiology

Hemisectioned slices were transferred to a chamber (RC-26; Warner Instruments) mounted to a fixed stage on a vibration isolation table (TMC Vibration Control). Slices were continuously perfused (3 ml/min) with oxygenated aCSF (126 mm NaCl, 3 mm KCl, 1.2 mm NaH_2_PO_4_, 26 mm NaHCO_3_, 11 mm glucose, 1.5 mm MgCl_2_, and 2.4 mm CaCl_2_ using a peristaltic pump; Cole-Parmer), and warmed to 30–32°C using an in-line solution heater (Warner Instruments). The identification of OFC^PV^ neurons in layer V of the ventrolateral OFC was achieved through excitation of the virally expressed enhanced yellow fluorescent protein (eYFP), using light from a mercury lamp (X-Cite 120Q, Excelitas) directed through the objective of an upright microscope (BX51WI, Olympus) mounted on a motorized moveable stage. The microscope was also equipped with differential interference contrast (DIC) optics, and a 900-nm infrared light source to identify living neurons in brain slices containing the OFC.

Whole-cell recording electrodes were fabricated using borosilicate pipette glass (Sutter Instruments, 1.5 mm O.D. × 0.86 mm I.D.) and a horizontal puller (P-97; Sutter Instruments). They were filled with a potassium-based internal solution (140 mm K-gluconate, 5 mm KCl, 10 mm HEPES, 0.2 mm EGTA, 2 mm MgCl_2_, 4 mm Mg-ATP, 0.3 mm Na_2_-GTP, and 10 mm Na_2_-phosphocreatine), neutralized to a pH of 7.2 using potassium hydroxide. Electrode resistances were 3–7MΩ when filled with this solution. Whole-cell patch clamp recordings were performed using an Molecular Devices 700B Multi-Clamp amplifier (Molecular Devices). Depending on the experiment, cells were voltage clamped between −50 and −65 mV. Spontaneous EPSCs (sEPSCs) were recorded in the presence of picrotoxin (50 μm) to block GABA_A_ receptor currents and sampled at 10 kHz using WinLTP software (WinLTP Ltd) and an A/D board (National Instruments, PCI-6251) housed in a personal computer. Analyses of sEPSCs were performed offline and automated using the MiniAnalysis program (v 6.0.7, Synaptosoft). The sEPSC detection parameters were as follows: amplitude threshold, 5 pA; period to search for peak, 10,000 μs; necessary baseline before peak, 5000 μs; period for decay, 20,000 μs; fraction of peak to find decay, 0.37; period of averaged baseline, 1000 μs; area threshold trigger, 20 pA/ms. Peak detection valence was 'negative', and all detected events were visually inspected to verify analysis. An examination data from randomly sampled sEPSC recordings indicated that RMS noise ranged from 1.86 to 2.88 pA, with a mean (±SEM) of 2.49 ± 0.88 pA (*n* = 9). This indicates adequate sensitivity to resolve sEPSCs above the 5-pA threshold. This same sample of cells showed a sEPSC mean 50% rise time of 0.33 ± 0.12 ms, and a 37% decay time of 0.97 ± 0.34 ms. Hyperpolarizing voltage steps (−10 mV) were delivered via the recording electrode every 30s to monitor whole-cell access and series resistance. Cells with an access resistance change of >30% were excluded from analysis. Current clamp experiments were performed with depolarizing currents ranging from −30 to 600 pA. ChR2 stimulation was achieved with blue laser light (473 nm; 50 mW laser, OEM) collimated through the objective using an IS-OGP adapter (Siskyou Corp.). Serotonin (5-HT, 1 mm; Tocris), was prepared fresh each day at low pH (∼2) to prevent oxidation and diluted 1:50 in aCSF flowing into the slice chamber, to achieve a final bath concentration of 20 μm using a syringe pump (Model A-99, Razel Scientific Instruments). All other drugs were dissolved at their final concentration in aCSF and delivered using the peristaltic pump. Light-evoked (optical) IPSCs (oIPSCs) were recorded in the presence of 6,7-dinitroquinoxaline-2,3-dione (DNQX; 20 μm) to block glutamatergic synaptic currents.

### Statistical analyses

Male and female Pvalb-iCre rats were randomly assigned to naive or CSA groups after intra-OFC injection of AAV5-EF1α-DIO-hChR2(H134R)-EYFP and recovery from this surgery. Formal experimenter blinding procedures were not used. However, complete analysis of the data and statistical comparisons from naive and CSA groups were not performed until data were collected from all subjects. Two rats were excluded from the studies, one because of the loss of intravenous catheter patency, the other died from an unknown cause during training. All experiments were designed using estimates of effect size and dispersion obtained from experience with similar OFC brain slice preparations. These estimates were then entered into power analysis calculations using G-Power (version 3.1.9.7, University of Dusseldorf, Germany) to ensure sample sizes were within reasonable estimates. In instances where experimental variability was large, additional experiments were performed. As stated above, recording from neurons exhibiting a change in access resistance (monitored continuously throughout the experiment) >30% were ceased and the data discarded. The proportion of cells discarded for this reason is estimated to be not >15%. Graphing and statistical analyses were performed using GraphPad Prism (v9.2). Data are generally reported as mean ± SEM or mean ± 95% confidence interval (CI), unless otherwise stated. Table 2 summarizes all statistical data, including tests of normality, significance levels, mean effect sizes, 95% CIs, and *post hoc* results (Tukey’s *post hoc* multiple comparisons test) for each experiment. Statistical tests included ANOVA [one-way, two-way, with repeated measures (RM) where appropriate], and the Student’s *t* test. The Fisher’s exact test was used to determine proportional effect changes among neurons from naive and CSA rats. A minimum level of *p* < 0.05 was considered to be significant in all statistical tests. Throughout, the number of rats from which data were acquired is indicated by *N*, whereas the number of cells is indicated by *n*.

**Table 2:** Statistical summary and analysis methods

Figure reported	*N*, # rats	*n*, # cells	Norm. dist?*	Statistic	Statistic value (df)	*p* value	Variance source	*Post hoc* test	*Post hoc p*	Mean difference	Lower 95% CI	Upper 95% CI
1*D*, mCherry vs GFP	6	N/A	N/A	Unpaired *t* test	*t*_(10)_ = 3.75	0.0038	Difference			36.4	14.75	58.04
												
3*A*, eYFP vs No eYFP (current inject vs eYFP)	11	23	Yes	Two-way RM-ANOVA	*F*_(11,231)_ = 30.23	<0.0001	Interaction			55.91	34.80	77.00
3*A*, eYFP+ vs eYFP (current inject)	11	23	Yes	Two-way RM-ANOVA	*F*_(11,231)_ = 51.63	<0.0001	Main effect					
3*A*, eYFP+ vs eYFP (eYFP)	11	23	Yes	Two-way RM-ANOVA	*F*_(1,21)_ = 17.61	0.00040	Main effect					
3*D*, eYFP+ vs eYFP (max current)	4	15	Yes	Unpaired *t* test	*t*_(22)_ = 5.804	<0.0001	Difference			82.03	55.61	108.50
4*B*, control	6	14	Yes									
4*B*, gabazine	6	14	Yes	Paired *t* test	*t*_(13)_ = 6.485	<0.0001	Difference			−101.90	−135.80	−67.95
5*A*, R_in_ (data not shown	11	28	Yes	Unpaired *t* test	*t*_(21)_ = 1.25	0.2250	Difference			102.60	98.25	107.00
5*A,B*, control	11	22		Unpaired *t* test	*t*_(80)_ = 0.38	0.0970	Difference			−0.104	−5.46	5.25
5*C*, outward I	11	4		One-sample test	*t*_(40)_ = 7.94	<0.0001	Difference			9.20	6.86	11.50
5*D*, ketanserin, male vs female	8	16	Yes	Two-way ANOVA	*F*_(1,27)_ = 8.671	0.0066	Interaction					
5*D*, ketanserin, male vs female	8	16		Two-way ANOVA	*F*_(1,27)_ = 8.135	0.0082	Main effect (sex)					
5*D*, ketanserin, male vs female	8	16		Two-way ANOVA	*F*_(1,27)_ = 3.694	0.0652	Main effect (treatment)					
5*D*, ketanserin, male vs female	8	16					Male vs female 5-HT	Tukey	0.880	5.30	−14.31	24.92
5*D*, ketanserin, male vs female	8	16					Male 5-HT vs +ketanserin	Tukey	>0.999	0.48	−21.17	22.13
5*D*, ketanserin, male vs female	8	16					Female 5-HT vs +ketanserin	Tukey	0.0007	−30.06	−48.41	−11.71
5*D*, ketanserin, male vs female	8	16					Female ketanserin vs Male ketanserin	Tukey	0.0116	−25.24	−45.75	−4.72
6*Bz*, sEPSC interevent male vs female	8	17	Yes	Kolmogorov–Smirnov	0.6455	<0.0001	Difference			177.7	168.00	187.30
6*D*, sEPSC frequency male vs female and 5-HT	8	17	Yes	Two-way ANOVA	*F*_(1,30)_ = 0.0402	0.842	Interaction					
6*D*, sEPSC frequency male vs female	8	17	Yes	Two-way ANOVA	*F*_(1,30)_ = 20.52	<0.0001	Main effect (sex)					
6*D*, sEPSC frequency 5-HT	8	17	Yes	Two-way ANOVA	*F*_(1,30)_ = 0.1990	0.6590	Main effect (5-HT)					
6*D*, sEPSC frequency male vs female	8	17					Male vs Female baseline sEPSC	Tukey	0.011	12.31	2.30	22.32
6*D*, sEPSC frequency male vs female and 5-HT	8	17					Male baseline vs male 5-HT	Tukey	0.970	1.68	−8.62	11.98
6*D*, sEPSC frequency male vs female and 5-HT	8	17					Female baseline vs female 5-HT	Tukey	0.998	0.64	−9.07	10.35
6*E*, sEPSC amplitude 5-HT	8	17	Yes	Two-way ANOVA	*F*_(1,30)_ = 0.0016	0.9680	Interaction					
6*E*, sEPSC amplitude 5-HT	8	17	Yes	Two-way ANOVA	*F*_(1,30)_ = 0.0085	0.9270	Main effect (sex)					
6*ED*, sEPSC amplitude 5-HT	8	17	Yes	Two-way ANOVA	*F*_(1,30)_ = 0.0054	0.9418	Main effect (5-HT)					
7*B*, 10-Hz train	8	21	N/A	Two-way ANOVA	*F*_(1,20)_ = 6.100	0.0226	Treatment			8.86	−26.95	44.67
7*B*, 20-Hz train	8	19	N/A	Two-way ANOVA	*F*_(1,18)_ = 4.618	0.0455	Treatment			3.97	−36.12	44.06
8*B*, total cocaine intake male-female	17	N/A		Unpaired *t* test	*t*_(22)_ = 1.463	0.1577	Difference			4.89	−2.04	11.82
8*C*, CSA, RMP	19	45	Yes	Two-way ANOVA	*F*_(1,35)_ = 13.26	0.0009	Interaction					
8*C*, CSA, RMP	19	45			*F*_(1,35)_ = 2.279	0.1401	Main effect (sex)					
8*C*, CSA, RMP	19	45			*F*_(1,35)_ = 4.459	0.0419	Main effect (treatment-CSA)					
8*C*, CSA, RMP	19	45	Yes				Naive male vs naive female	Tukey	0.520	−4.50	−13.30	4.30
8*C*, CSA, RMP	19	45	Yes				Naive male vs CSA male	Tukey	0.0015	−12.15	−20.26	−4.03
8*C*, CSA, RMP	19	45					CSA male vs CSA female	Tukey	0.0014	10.88	3.646	18.10
8*C*, CSA, RMP	19	45					Naive female vs CSA female	Tukey	0.698	3.23	−4.763	11.22
8*D*, CSA, I-Hold	30	72	Yes	Two-way ANOVA	*F*_(1,68)_ = 9.115	0.0036	Interaction					
8*D*, CSA, I-Hold	30	72			*F*_(1,68)_ = 0.0036	0.9521	Main effect (sex)					
8*D* , CSA, I-Hold	30	72			*F*_(1,68)_ = 7.145	0.0094	Main effect (treatment-CSA)					
8*D*, CSA, I-Hold	30	72					naive male vs naive female	Tukey	0.261	35.51	−15.164	86.19
8*D*, CSA, I-Hold	30	72					Naive male vs CSA male	Tukey	<0.0001	68.32	29.495	107.15
8*D*, CSA, I-Hold	30	72					Naive female vs CSA female	Tukey	0.996	−4.15	−54.050	45.74
8*D*, CSA, I-Hold	30	72					CSA male vs CSA female	Tukey	0.057	−36.96	−74.761	0.84
8*E*, firing, male CSA	8	17	Yes	One-way RM-ANOVA	*F*_(7,21)_ = 1.288	0.3047	Treatment			−2.421	−23.500	18.70
8*F*, firing, female CSA	11	24	Yes	One-way RM-ANOVA	*F*_(7,21)_ = 1.288	0.3047	Treatment			6.67	−9.047	22.36
8*G*, Ihold 5-HT, CSA, male	16	39	N/A	Unpaired *t* test	*t*_(38)_ = 0.6226	0.5373	Treatment			−1.625	−6.909	3.66
8*H*, Ihold 5-HT, CSA, female	14	30	N/A	Unpaired *t* test	*t*_(38)_ = 0.0742	0.9412	Treatment			−0.290	−8.154	7.58
8, data (male female proportions)	30	66	N/A	Fisher’s	N/A	0.7021	Treatment			N/A	N/A	N/A
8 data (male female days CSA withdrawal)	30	66	Yes	Unpaired *t* test	*t*_(17)_ = 1.733	0.1013	Sex			18.82	−4.098	41.74
9*B*, sEPSC frequency male vs female CSA	19	43	Yes	Two-way ANOVA	*F*_(1,39)_ = 12.05	0.0013	Interaction					
9*B*, sEPSC frequency male vs female CSA	19	43			*F*_(1,39)_ = 4.434	0.0417	Main effect (sex)					
9*B*, sEPSC frequency male vs female CSA	19	43			*F*_(1,39)_ = 2.057	0.1595	Main effect (treatment-CSA)					
9*B*, sEPSC frequency male vs female CSA	19	43					Naive male vs naive female	Tukey	0.0047	12.31	3.137	21.49
9*B*, sEPSC frequency male vs female CSA	19	43					Naive male vs CSA male	Tukey	0.006	10.83	2.563	19.10
9*B*, sEPSC frequency male vs female CSA	19	43					Naive female vs CSA female	Tukey	0.494	−4.50	−12.984	3.99
9*B*, sEPSC frequency male vs female CSA	19	43					CSA male vs CSA female	Tukey	0.704	−3.02	−10.510	4.48
9*C*, sEPSC amplitude male vs female CSA	19	42	Yes	Two-way ANOVA	*F*_(1,38)_ = 0.0691	0.8018	Interaction					
9*C*, sEPSC amplitude male vs female CSA	19	42			*F*_(1,38)_ = 0.1930	0.6629	Main effect (sex)					
9*C*, sEPSC amplitude male vs female CSA	19	42			*F*_(1,38)_ = 1.998	0.1657	Main effect (treatment-CSA)					
9*D*, sEPSC frequency male vs female CSA, 5-HT	19	42	Yes	Two-way ANOVA	*F*_(1,38)_ = 0.5817	0.4503	Interaction					
9*D*, sEPSC frequency male vs female CSA, 5-HT	19	42			*F*_(1,38)_ = 0.3550	0.5548	Main effect (sex)					
9*D*, sEPSC frequency male vs female CSA, 5-HT	19	42			*F*_(1,38)_ = 0.095	0.7600	Main effect (treatment-CSA)					

* D’Agostino–Pearson test of normality.

## Results

### Generation of transgenic rats and their characterization

A BAC DNA containing the rat Pvalb coding region was successfully recombineered to contain iCre at the start codon of the Pvalb coding sequence (Fig. 1*A*). The resulting BAC was injected into rat embryos and resulted in three founder lines. One founder line did not propagate. Phenotyping animals from the other two lines was conducted by Cre-mediated recombination of an AAV vector expressing a nuclear-localized, color-changing reporter, AAV-Nuc-flox-(mCherry)-eGFP (Bäck et al., 2019). Line 2 exhibited Cre-recombination in the OFC (Fig. 1*B–D*). The number of mCherry-positive cells (no recombination) was clearly higher than the sparse number of eGFP-positive cells (Cre recombination). Quantification revealed the percentage of eGFP-positive cells coexpressing parvalbumin was variable but much higher than cells coexpressing mCherry and parvalbumin (Fig. 1*D*). The lack of parvalbumin immunoreactivity in some eGFP expressing cells may be because of fluctuations in parvalbumin expression, and therefore Cre, relative to when the injection occurred. The ability to colocalize parvalbumin signal with nuclear fluorescent signal may also be a limiting factor. Importantly, cells that showed no signs of recombination (mCherry positive cells) consistently showed minimal overlap with parvalbumin expression (Fig. 1*B*,*D*). Using AAV with Cre-dependent reporters in this manner, we followed several potential sublines over 3–4 generations showing Cre-recombination in areas where Pvalb is known to be expressed, such as prefrontal and somatosensory cortices, with a focus on the OFC. We also observed a subset of neurons in both somatosensory cortex and non-dopaminergic neurons in the midbrain that exhibited Cre activity. Moreover, another laboratory characterizing this same line of rats found that 98% of cells expressing a Cre-dependent eYFP in the amygdala also expressed parvalbumin immunoreactivity (unpublished observation; Gavan McNally, personal communication). Using another Cre-dependent, AAV vector expressing a translational fusion of the rhodopsin ion channel to eYFP (AAV-DIO- hChR2-eYFP), we also examined colocalization with endogenous Pvalb expression in the OFC (Fig. 1*E–J*). Cells with somal expression of ChR2-eYFP colocalized with Pvalb immunofluorescence. The cellular pattern of Pvalb-immunofluorescence was similar between ChR2-eYFP-positive cells at the injection sight and ChR2-eYFP-negative cells distal to the site of AAV-DIO-hChR2-eYFP injection indicating the transgene expression did not alter Pvalb expression. As with our injection of the AAV Nuc flox (mCherry)eGFP, we noted cells expressing hChR2-eYFP but not Pvalb, suggesting the presence of low level non-specific Cre expression or that the hChR2-eYFP expressing cells were no longer producing Pvalb at the time of tissue collection. Other brain regions were not extensively characterized in this line of transgenic rats, and any Cre-dependent transgene of interest should be empirically evaluated for a given brain area and testing paradigm. Based on our phenotypic data and finding that only a single copy of the Cre transgene was present in line 2, we designated the line as LE-Tg(Pvalb-iCre)2Ottc [rat genome database (RGD) ID #10412329, RRRC line #773] and refer to this line as Pvalb-iCre for the remainder of the article.

#### Properties of transfected OFC^PV^ neurons

**Figure 2.**
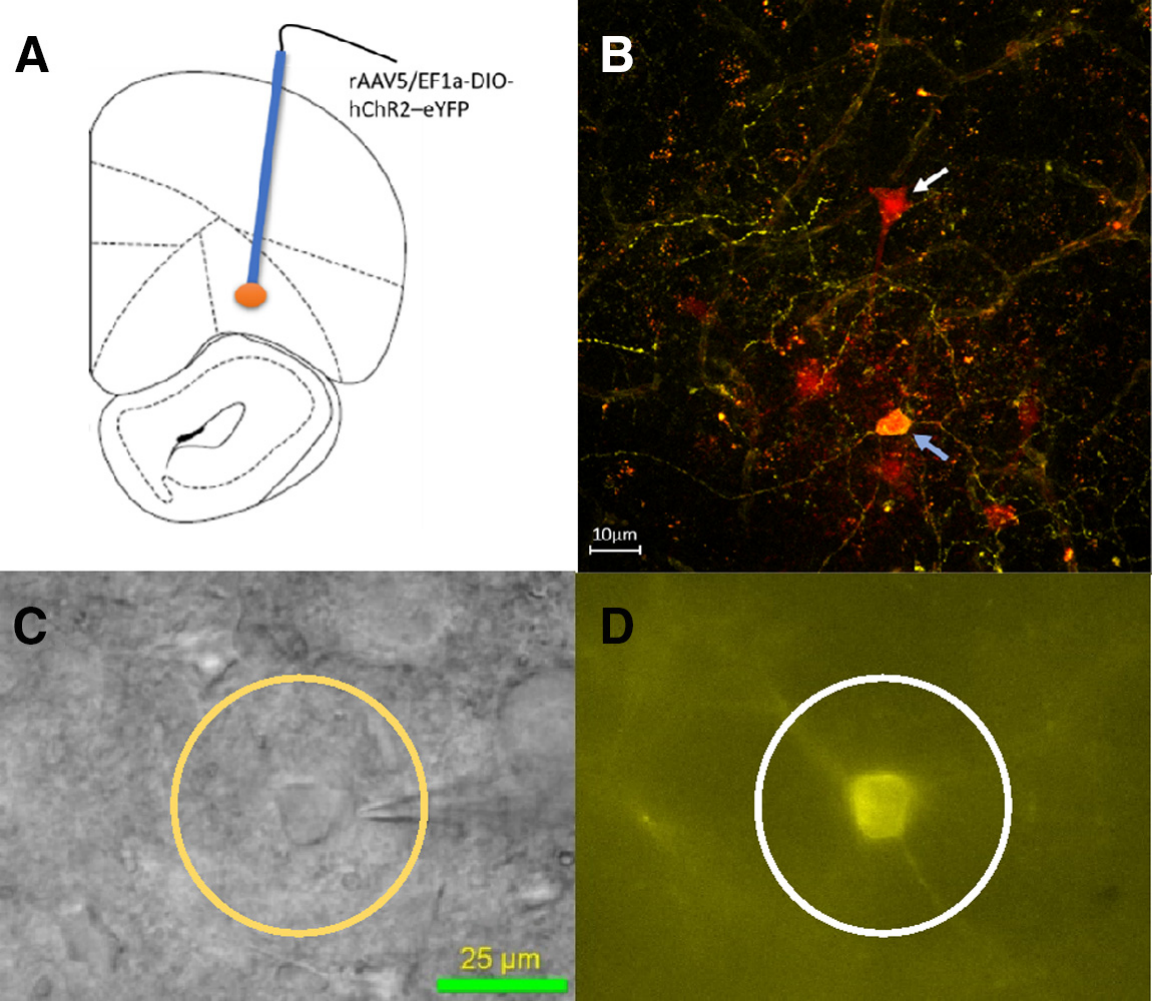
***A***, Schematic illustration of virus injection site in the lateral OFC (+4.0 mm anterior of Bregma; –2.0 mm from midline, −4.3 mm ventral to skull surface; adapted from Paxinos and Watson, 1998). ***B***, OFC PyN filled with biocytin bound to an Alexa Fluor 594 conjugate (white arrow). Also shown is a biocytin-filled cell that also expresses eYFP (blue arrow) in a Pvalb-iCre rat, three weeks after receiving an OFC injection with the ChR2-eYFP viral construct. ***C***, DIC video microscopic image showing an OFC neuron (yellow circle) in a living rat brain slice from a Pvalb-iCre rat. A patch electrode is shown to the right (v-shaped structure). ***D***, The same neuron (white circle) as in ***C***, under fluorescence illumination, and through the same objective in a Pvalb-iCre rat, three weeks after injection with the ChR2-eYFP virus (scale bar for ***C***, ***D***).

**Figure 3.**
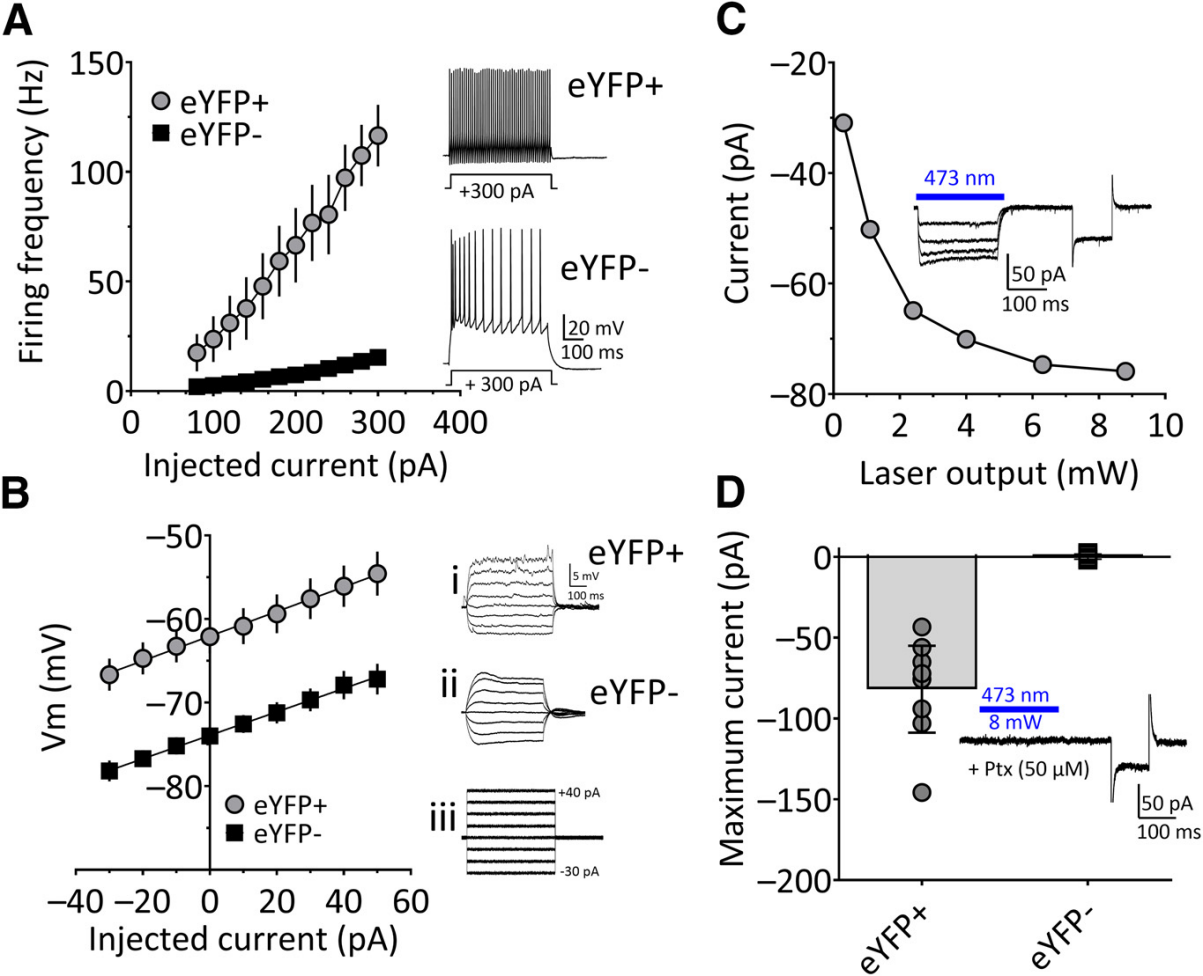
Properties of eYFP-positive (eYFP+) and eYFP-negative (eYFP–) neurons in the OFC of Pvalb-iCre rats, three weeks after injection of viral construct expressing eYFP and ChR2. ***A***, left, Mean effect of depolarizing currents (80–300 pA, 500 ms), passed through whole-cell recording electrodes, on action potential firing frequency in eYFP+ and eYFP– cells. eYFP+ cells showed a significantly higher frequency of action potential discharge, compared with eYFP– cells (significant interaction between eYFP and current injection; *F*_(11,231)_ = 30.23, two-way RM ANOVA). Inset, Representative examples from single cells responding to +300-pA current injection. ***B***, Mean I–V relationships in response to subthreshold current injections (–30 to +50 pA, 500 ms) in eYFP+ and eYFP– cells. Membrane potential measurements were made during the final 20 ms of the current step. i–iii show membrane voltage responses to current injection in representative eYFP+ and eYFP– neurons, as well as the current steps to evoke these responses (iii). ***C***, Peak inward currents elicited by ChR2 activation with 473-nm laser light pulses of varying power in a single eYFP+ OFC neuron. The inset shows membrane currents in response to different intensities of 473-nm light (blue bar). The later current response is used to measure R_in_. The cell was voltage clamped at −65 mV. ***D***, Mean ± 95% CI maximum inward currents indicating that light-evoked responses were observed in eYFP+ cells, and not in eYFP– cells (*N*, *n* = 4, 15; unpaired *t* test, *t*_(22)_ = 5.804, *p* < 0.0001). In these experiments, GABAergic currents were blocked by picrotoxin (Ptx; 50 μm) to eliminate IPSCs arising from activation of nearby eYFP+ cells.

Following delivery of AAV expressing Cre-dependent ChR2-eYFP, we confirmed eYFP fluorescence in OFC and used DIC microscopy to locate cells in spatial register with the fluorescent signal (Fig. 2). Patch clamp recordings in eYFP-positive neurons revealed a significantly higher firing frequency in response to square-wave current injection, compared with eYFP-negative cells (significant interaction between eYFP expression and current injection, two-way RM-ANOVA; *F*_(11,231)_ = 30.23, *p* < 0.0001; and significant main effects of firing frequency vs injected current (*F*_(11,231)_ = 51.63, *p* < 0.0001) and firing rate as a function of YFP expression (*F*_(1,21)_ = 17.61, *p* = 0.0004; Fig. 3*A*). The eYFP-positive neurons also exhibited more depolarized resting membrane potentials (RMPs) than eYFP-negative neurons (Fig. 3*B*), and injection of subthreshold depolarizing current revealed current oscillations in eYFP-positive cells (Fig. 3*Ci*), that were not seen in eYFP-negative cells (Fig. 3*Cii*). In addition, the current–voltage (I–V) relationships in eYFP-positive cells were linear (Fig. 3*B*, deviation from linearity, *p* = 0.26), unlike the profile seen in other interneuron subtypes. As the electrophysiological characteristics observed in eYFP-positive cells matched that of fast-spiking parvalbumin interneurons (Koós and Tepper, 1999; Weber and Andrade, 2010), we refer to these eYFP-positive cells in our Pvalb-iCre rats as OFC^PV^ neurons.

#### ChR2-induced activity in OFC^PV^ cells

**Figure 4.**
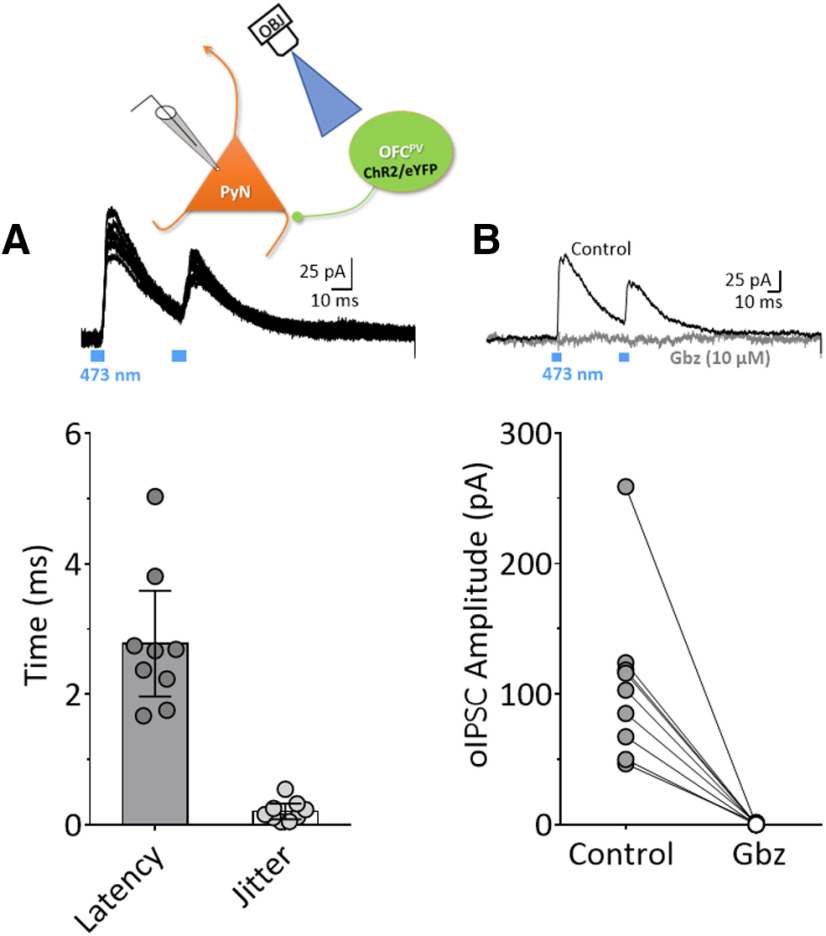
Optogenetic stimulation of OFC^PV^ cells evokes monosynaptic IPSCs in OFC PyNs. The diagram indicates the recording and light-stimulation configuration. Recordings were made in OFC PyNs and stimulation of ChR2 expressed in OFC^PV^ neurons with blue light passed through the microscope objective (OBJ). ***A***, top panel, Individual traces of paired (50-ms interval) optical IPSCs (oIPSCs) evoked by stimulation ChR2 with 473-nm light, recorded in an eYFP– cell. Bottom panel, Individual and mean (±95% CI) peak first response to light stimulation showing latency to 50% peak oIPSC (2.78 ± 1.05 ms) and jitter (0.20 ± 0.16 ms). ***B***, top panel, Representative sample of a paired synaptic response to 473-nm stimulation that is eliminated by the GABA_A_ receptor antagonist gabazine (Gbz; 10 μm, gray trace). Bottom panel, Summary of first oIPSC amplitudes recorded from OFC PyNs demonstrating complete elimination of the response by Gbz (*N*, *n* = 6, 14, paired *t*_(13)_ = 6.49, *p* < 0.0001).

Recorded OFC^PV^ cells were stimulated using blue (473 nm) laser light across a range of power settings (200 ms in duration). Laser stimulation generated stable inward currents without decrement throughout the light pulse, at all power settings (Fig. 3*C*). In contrast, currents were not observed in eYFP-negative cells in response to laser stimulation (Fig. 3*D*, *t*_(13)_ = 6.71, *p* < 0.0001). In a group of recordings from PyNs (eYFP-negative cells), we found that single 5 ms laser pulses reliably elicited IPSCs (oIPSCs; Fig. 4*A*). These oIPSCs had a short (2.78 ± 1.05 ms) latency to 50% peak with a small SD (0.20 ± 0.16 ms, mean ± SD) or “jitter”; both of which suggest that these currents were monosynaptic (Fig. 4*A*). Paired-pulse laser stimulation at a 50-ms interpulse intervals revealed inhibition of the second oIPSC, relative to the first oIPSC (Fig. 4*A*), and these responses were completely blocked by the selective GABA_A_ receptor antagonist, SR-95531 (gabazine, 10 μm; Fig. 4*B*, paired-Student’s *t* test, *t*_(13)_ = 6.49, *p* < 0.0001). Thus, ChR2 activation of OFC^PV^ interneurons generates GABAergic inhibition of OFC PyNs in Pvalb-iCre rats.

##### 5-HT Effects in OFC^PV^ cells

**Figure 5.**
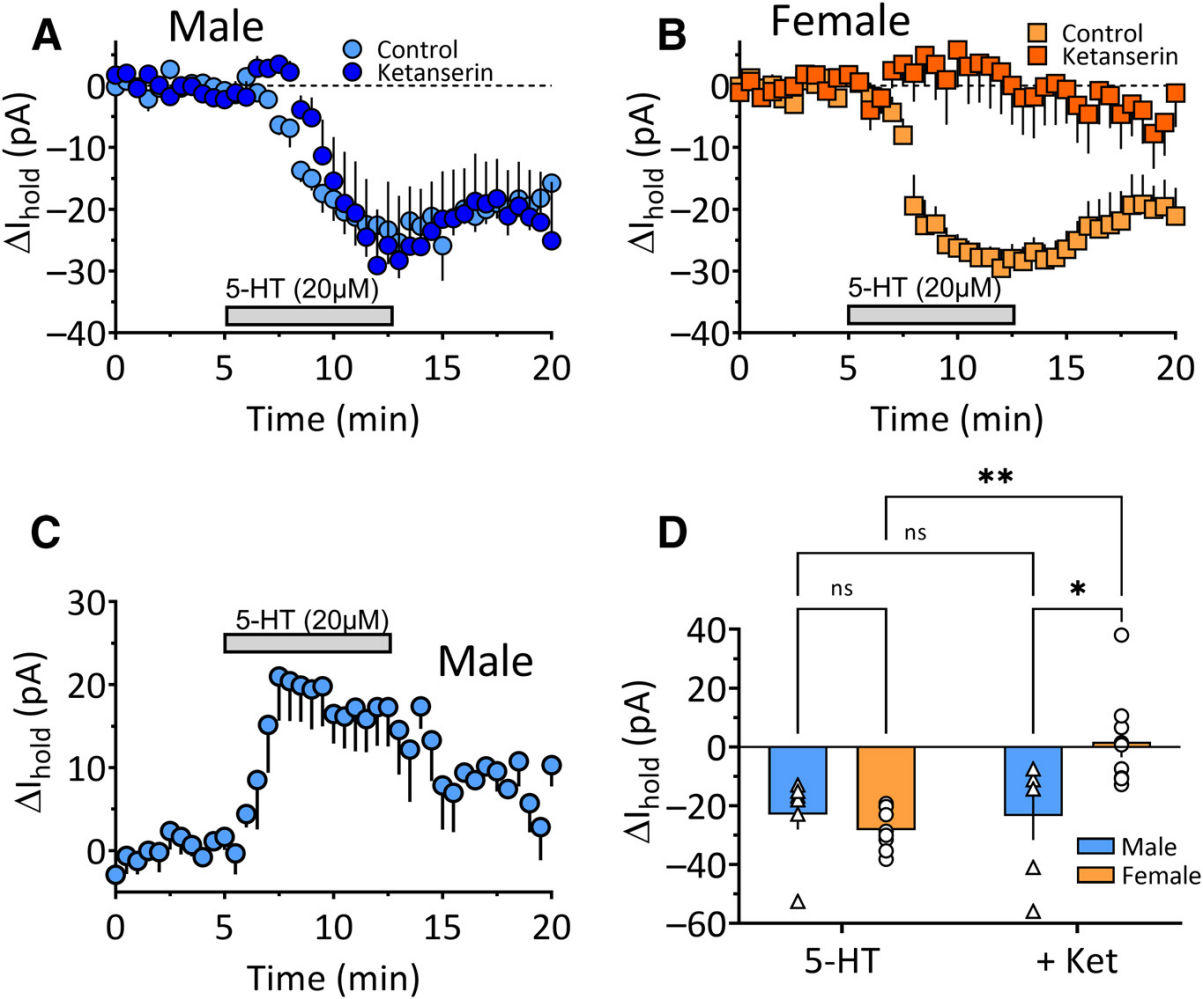
Effects of 5-HT and the 5-HT_2A/2C_ antagonist ketanserin (Ket; 10 μm) on OFC^PV^ cell I_holds_ in cocaine-naive rats. ***A***, Time course of mean (±SEM) inward currents caused by bath application of 5-HT (20 μm, horizontal gray bar) alone, or during treatment with ketanserin in male Pvalb-iCre rats (*N*, *n* = 7, 13). Inward currents with 5-HT alone were observed in the majority of OFC^PV^ neurons in males and females (22/28 cells, 11 rats, 78.6%). ***B***, Time course of mean (±SEM) inward currents caused by bath application of 5-HT alone, or during application of ketanserin in female Pvalb-iCre rats (*N*, *n* = 4, 11). ***C***, Mean (±SEM) outward 5-HT-induced currents were observed in a minority of male OFC^PV^ cells (4/28 cells, 14.3%). ***D***, Mean maximal effects of 5-HT alone, or during application of ketanserin in OFC^PV^ neurons from male and female Pvalb-iCre rats. The effects of 5-HT under control and ketanserin conditions were obtained by averaging data across the final 3 min of the 5-HT application periods shown in ***A***, ***B***. Note that although the magnitudes of the 5-HT-induced inward currents did not differ between male and female OFC^PV^ neurons in the control condifontstion, they were significantly blocked by ketanserin only in female Pvalb-iCre rats (two-way ANOVA, sex × treatment interaction *F*_(1,27)_ = 8.671, *p* = 0.0066; Tukey’s *post hoc* comparisons, male 5-HT vs ketanserin *p* > 0.9999, female 5-HT vs ketanserin, **p = 0.0007, male vs female ketanserin, *p = 0.0116). ns, not significant.

Bath application of 5-HT (20 μm) elicited inward currents at a holding potential of –65 mV in the majority of OFC^PV^ cells examined (22 of 28 cells, 11 rats, 78.6%; Fig. 5*A*,*B*), with no change in input resistance (R_in_; data not shown; one-sample *t*_(21)_ = 1.25, *p* = 0.225). Moreover, the mean largest amplitudes of inward currents in response to 5-HT in OFC^PV^ neurons from males and female rats were similar (Fig. 5*A–C*, unpaired *t* test, *t*_(80)_ = 0.38, *p* = 0.97). A small number of cells (4/28, 14.3%) from only male rats exhibited significant outward currents in response to 20 μm 5-HT (Fig. 5*C*, one-sample *t* test, *t*_(40)_ = 7.94, *p* < 0.0001), and this was associated with a small decrease in R_in_ (data not shown). Given the small proportion of cells showing outward currents, we did not further characterize this response. However, it has previously been shown that similar currents in OFC PyNs in male wild-type rats were mediated by 5HT_1A_ receptors (Wright et al., 2017).

In OFC^PV^ neurons from female rats, inward currents were blocked by pretreatment of brain slices with ketanserin (10 μm), a 5-HT_2A/2C_ receptor antagonist (Fig. 5*B*,*D*), but ketanserin did not change the peak effect of 5-HT in OFC^PV^ neurons from male rats (Fig. 5*A*,*D*, two-way ANOVA sex × treatment interaction, *F*_(1,27)_ = 8.67, *p* = 0.0066, main effect of sex, *F*_(1,27)_ = 8.14, *p* = 0.0082, main effect of treatment, *F*_(1,27)_ = 3.70, *p* = 0.0652). Moreover, although *post hoc* analysis (Tukey) indicated no difference between males and females with regard to the basal effect of 5-HT on inward currents in OFC^PV^ neurons (*p* = 0.881), there was a significant effect of ketanserin in females (*p* = 0.0007), but not in males (*p* > 0.999).

#### 5-HT does not alter glutamatergic transmission onto OFC^PV^ cells

**Figure 6.**
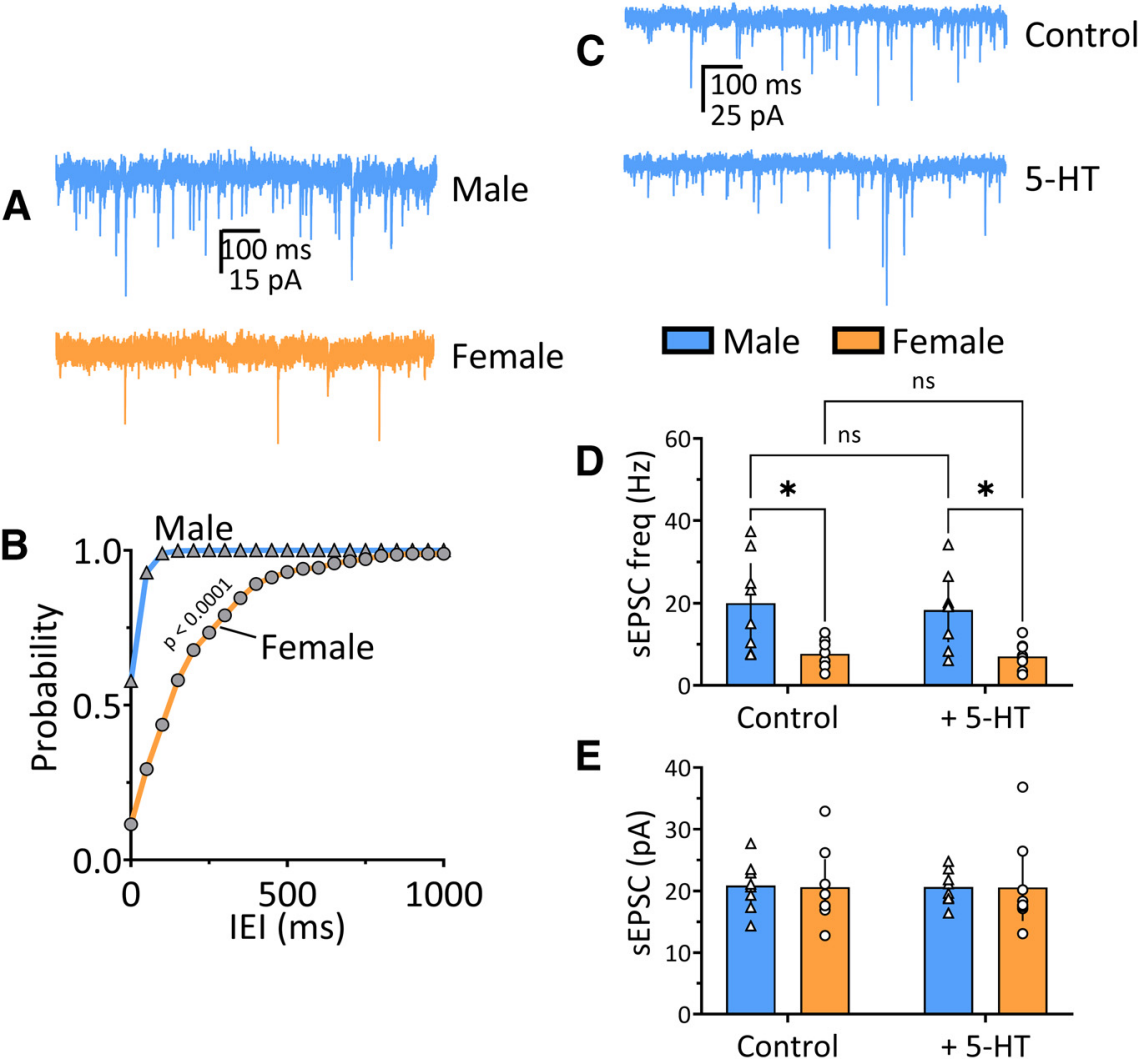
Excitatory transmission onto OFC^PV^ cells differs between male and female rats and is insensitive to 5-HT. ***A***, Representative traces of sEPSCs from male and female OFC^PV^ cells from Pvalb-iCre rats. ***B***, Mean cumulative probability histogram of interevent intervals (IEIs) showing significantly (*p* < 0.0001, Kolmogorov–Smirnov test) larger intervals between sEPSC in OFC^PV^ cells from naive female rats, compared with males. ***C***, Representative sEPSC traces in an OFC^PV^ neuron from a male rat before (control) and during 5-HT (20 μm) application. ***D***, Mean (±95% CI) sEPSC frequency recorded in OFC^PV^ neurons in male and female Pvalb-iCre rats. sEPSC frequency was significantly lower in cells from female rats (significant main effect of sex, *F*_(1,30)_ = 20.52, *p* < 0.0001, two-way ANOVA; *p* = 0.011, Tukey’s *post hoc* test), but 5-HT application did not significantly change sEPSC frequency in males or females (main effect of treatment, *F*_(1,30)_ = 0.199, *p* = 0.66, two-way ANOVA; *p* = 0.011, *p* = 0.97, and *p* = 0.99, respectively, Tukey’s *post hoc* test). *p < 0.05, Tukey’s post-hoc test. ***E***, Mean (±95% CI) sEPSC amplitudes recorded in OFC^PV^ neurons in male and female *Pvalb-iCre* rats during baseline, and 5-HT application periods. The sEPSC amplitudes did not differ between male and female rats, and there were no significant effects of 5-HT on sEPSC amplitude (*F*_(1,30)_ = 0.00164, *p* = 0.97, two-way ANOVA). ns, not significant.

In addition to its postsynaptic effects, 5-HT increases glutamatergic transmission onto OFC PyNs, likely via depolarization of these cells and release of glutamate from PyN to PyN collateral synapses. We therefore examined properties of glutamate synaptic transmission onto OFC^PV^ cells, and whether this was modulated by 5-HT, by recording sEPSCs in these neurons. An interaction between sex and treatment (5-HT effect) on sEPSC frequency was not significant using a two-way ANOVA (*F*_(1,30)_ = 0.040, *p* = 0.842; Fig. 6*A–D*), nor was there a significant main effect of treatment with 5-HT on sEPSC frequency (*F*_(1,30)_ = 0.200, *p* = 0.658). However, there was a significant main effect of sex on the frequency of sEPSCs (*F*_(1,30)_ = 20.52, *p* < 0.0001). *Post hoc* analysis revealed that the frequency of sEPSCs were significantly higher in OFC^PV^ cells from males in the baseline condition (*p* = 0.0113, Tukey’s *post hoc* test) and that 5-HT had no effect of sEPSC frequency in these cells from males or females (*p* = 0.970, *p* = 0.997, respectively). In contrast to this baseline difference in sEPSC frequency between males and females, mean sEPSC amplitudes were not significantly different (Fig. 6*E*), nor did 5-HT alter this measure (Fig. 6*E*, two-way ANOVA, *F*_(1,30)_ = 0.00164, *p* = 0.97). Thus, basal synaptic glutamate input is stronger in male rat OFC^PV^ neurons, and this is not altered by 5-HT in males or females.

#### 5-HT increases GABAergic transmission onto OFC PYRs

**Figure 7.**
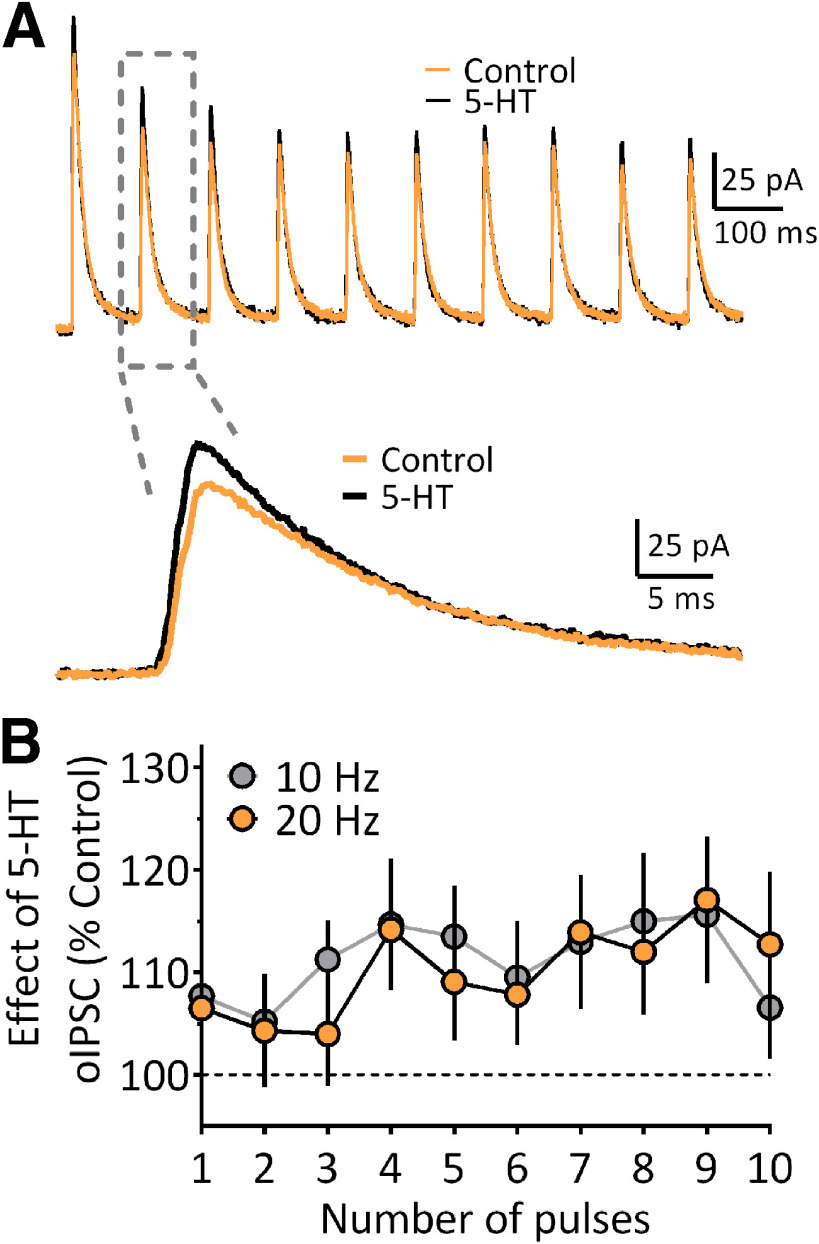
Effects of 5-HT on trains of oIPSCs recorded in OFC PyNs following light-activation of OFC^PV^ cells. The recording configuration is the same as described in Figure 4. ***A***, Traces of oIPSCs obtained from an OFC PyN before (control, orange line) and during 5-HT (20 μm) application (black line). The lower traces are those shown in the gray dashed box, plotted on an expanded timescale. ***B***, Mean (±SEM) of the effect of 5-HT on trains of oIPSCs evoked at 10 Hz (*n* = 21 cells) or 20 Hz (*n* = 19 cells), expressed as a percentage of control responses recorded before 5-HT-application. The oIPSCs activated at both frequencies were significantly increased by 5-HT (RM two-way ANOVA; 10-Hz train, treatment effect = *F*_(1,20)_ = 6.1, *p* = 0.023; 20-Hz train, treatment effect = *F*_(1,18)_ = 4.618, *p* = 0.045).

As shown above, 5-HT evoked inward depolarizing currents in the majority of GABAergic OFC^PV^ neurons, and these cells are synaptically connected to OFC PyNs (Figs. 4, 5). Therefore, we hypothesized that 5-HT would increase ChR2-evoked GABAergic oIPSCs recorded in OFC PyNs. Since parvalbumin interneurons can sustain high rates of firing, we activated OFC^PV^ cells with 473-nm light stimulus trains (10 pulses at 10 or 20 Hz; laser power set to evoke oIPSCs at 75% of maximum) to activate oIPSCs in PyNs. 5-HT (20 μm) significantly increased the amplitude of oIPSCs between ∼5% and 16% for the 10-Hz trains and 5% and 17% for the 20-Hz trains (Fig. 7*A*,*B*, RM two-way ANOVA; 10-Hz train = *F*_(1,20)_ = 6.1, *p* = 0.023; 20-Hz train = *F*_(1,18)_ = 4.618, *p* = 0.045). Roughly half (*N* = 8 rats, 11 of 21 cells, 52.4%) of the cells demonstrated an increase in oIPSCs in response to 5-HT, while a few (four of 21 cells, 19.0%) showed a decrease. The remaining cells (6 of 21, 28.6%) showed no change on 5-HT application. There were no differences in the magnitude of the 5-HT-induced increase in oIPSC amplitude in OFC^PV^ cells between male and female rats (data not shown), nor in the proportion of cells showing the 5-HT increase in oIPSCs. Together, these data suggest that 5-HT increases OFC^PV^ neuron inhibition of OFC PyNs and that this does not differ between male and female rats.

##### Effects of CSA withdrawal on OFC^PV^ neurons

**Figure 8.**
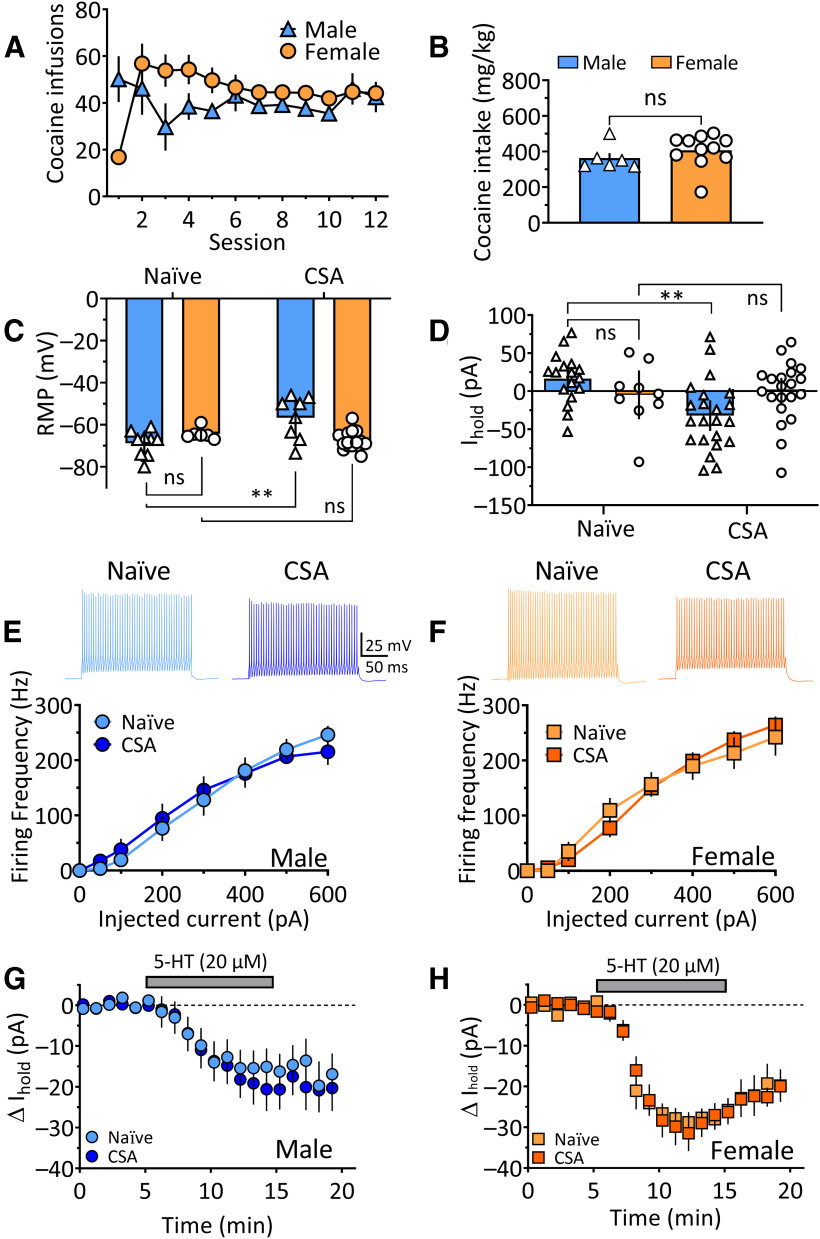
OFC^PV^ interneuron membrane properties and sensitivity to 5-HT following CSA in male and female Pvalb-iCre rats. ***A***, Mean number of lever presses for cocaine in male and female Pvalb-iCre rats. ***B***, Mean cocaine intake for all Pvalb-iCre rats across all self-administration trials. Male and female rats self-administered a similar amount of cocaine (unpaired *t* test, *t*_(22)_ = 1.46, *p* = 0.158). Legend applies to ***C***, ***D***. ***C***, Mean (±95% CI) RMP of OFC^PV^ neurons from naive male and female rats and those withdrawn from CSA. Cells from male rats were significantly depolarized (less negative RMP) following CSA withdrawal (two-way ANOVA, sex × treatment interaction, *F*_(1,35)_ = 13.26, *p* = 0.0009); male naive versus male CSA (**Tukey’s *post hoc* test, *p* = 0.0015), whereas those from females were unchanged (ns, *p* = 0.70, Tukey’s *post hoc* test). **p < 0.01, Tukey’s post-hoc test. ***D***, Mean (±95% CI) I_hold_ necessary to voltage clamp OFC^PV^ cells at −65 mV. Consistent with the changes in RMP, cells from male rats required more I_hold_ after CSA (two-way ANOVA, sex × treatment interaction, *F*_(1,68)_ = 9.115, *p* = 0.0036; male naive vs male CSA, *p* <0.0001, Tukey’s *post hoc* test), and cocaine exposure did not change I_hold_ in OFC^PV^ neurons from female rats (female naive vs female CSA, *p* = 0.9962, Tukey’s *post hoc* test). **p < 0.01, Tukey’s post-hoc test. ***E***, ***F***, Withdrawal from CSA did not alter the action potential discharge frequency of OFC^PV^ cells caused by depolarizing current injection in either males or females (two-way ANOVA, *F*_(7,21)_ = 1.288, *p* = 0.3047). ***G***, ***H***, Mean time course of the effects of 5-HT on I_hold_ in OFC^PV^ neurons from male and female naive and CSA rats. No changes in the sensitivity of OFC^PV^ neurons to 5-HT were observed in cells from males (unpaired *t* test, *t*_(38)_ = 0.6226, *p* = 0.5373) or females (*t*_(38)_ = 0.0742, *p* = 0.9412) following withdrawal from CSA. ns, not significant.

Although small differences in the rates of lever pressing were noted between male and female Pvalb-iCre rats during earlier sessions of acquisition of CSA, this became similar and stable in both sexes by the end of training (Fig. 8*A*). Moreover, there was no difference in the total amount of cocaine intake between male and female rats at the end of this training (Fig. 8*B*, *t*_(15)_ = 1.02, *p* > 0.05, unpaired *t* test).

#### OFC^PV^ neuron membrane properties are altered after withdrawal from CSA

Following 14 d of CSA training, rats were returned to their home cages for 55.7 ± 5.7 d (mean ± SEM) until electrophysiology experiments were conducted. Male and female rats were withdrawn from CSA for a similar number of days (45.8 ± 4.6 and 64.6 ± 9.4 d, respectively, *t*_(17)_ = 1.733, *p* = 0.101, unpaired *t* test). RMP, holding current (I_hold_; obtained at −65 mV) and R_in_, were measured in OFC^PV^ cells from naive rats and those withdrawn from CSA. Cells from male, but not female animals, demonstrated significantly less negative (depolarized) RMPs after withdrawal from CSA (Fig. 8*C*, two-way ANOVA, treatment × sex interaction, *F*_(1,35)_ = 13.26, *p* = 0.0009; treatment main effect *F*_(1,35)_ = 4.459, *p* = 0.0419; sex main effect, *F*_(1,35)_ = 2.279, *p* = 0.1401) *post hoc* analysis (Tukey’s) showed that there was no difference in OFC^PV^ RMPs in between naive females and males (*p* = 0.520), that there was a significant effect of CSA on RMP in only males (*p* = 0.0015) demonstrated by a significant difference between females and males after CSA (*p* = 0.0014). Consistent with the change in OFC^PV^ RMP values from male rats, the amount of current necessary to voltage clamp these cells at −65 mV (I_hold_) was larger and more negative in OFC^PV^ neurons from male rats after withdrawal from CSA (Fig. 8*D*, two-way ANOVA, treatment × sex interaction, *F*_(1,68)_ = 9.115, *p* = 0.0036; treatment main effect, *F*_(1,68)_ = 7.145, *p* = 0.0094; sex main effect, *F*_(1,68)_ = 0.0036, *p* = 0.9521). *Post hoc* analysis showed that the effect of CSA on I_hold_ in males was significant (*p* < 0.0001). In contrast to the effects of CSA on RMP and I_hold_ in OFC^PV^ neurons from male rats, no changes in the relationship between injected current and firing frequency was observed in any of the groups after CSA (Fig. 8*E*,*F*, RM-ANOVA, *F*_(7,21)_ = 1.288, *p* = 0.304). These data suggest that whereas OFC^PV^ neurons from only male rats are more depolarized following CSA, this has little effect on neuronal output in response to depolarizing current injection.

##### Effects of 5-HT on OFC^PV^ neurons after withdrawal from CSA

Withdrawal from cocaine did not affect the magnitude of mean change in I_hold_ caused by 5-HT (20 μm) in OFC^PV^ neurons from male or female Pvalb-iCre rats after withdrawal from CSA (Fig. 8*G*,*H*, unpaired *t* test, male *t*_(38)_ = 0.623, *p* = 0.5373; female *t*_(38)_ = 0.0742, *p* = 0.941). Furthermore, the proportion of cells showing inward currents with 5-HT was not changed after withdrawal from CSA (Fisher’s exact test, *p* = 0.702).

**Figure 9.**
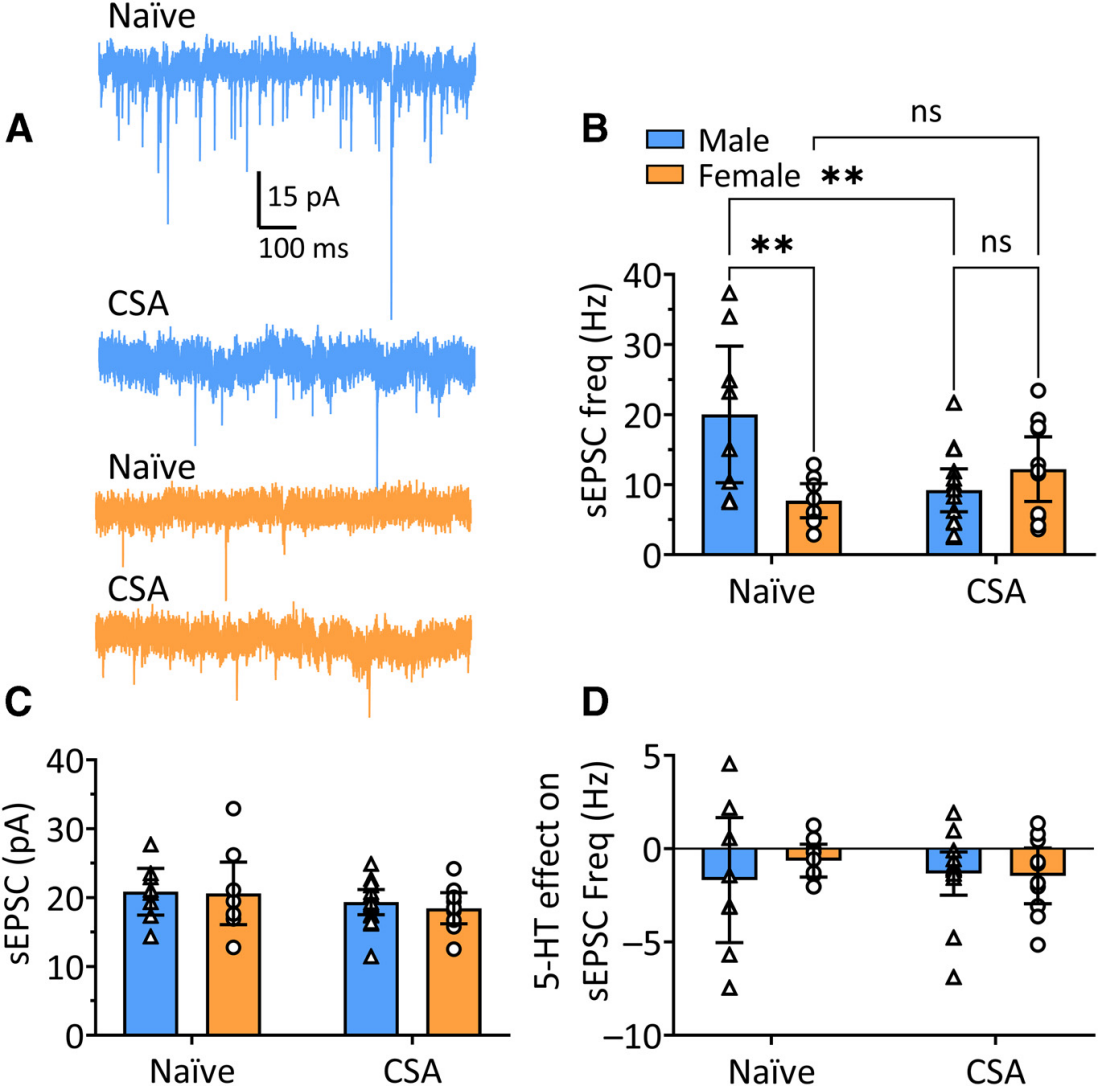
Properties of sEPSCs in OFC^PV^ interneurons following CSA and lack of effects of 5-HT in Pvalb-iCre rats. ***A***, Representative traces showing sEPSCs from male (blue traces) and female (orange traces) OFC^PV^ cells from naive rats and those withdrawn from CSA. ***B***, Mean (±95% CI) sEPSC frequency in OFC^PV^ cells from male and female naive rats and those withdrawn from CSA. There was a significant reduction in sEPSC frequency in male OFC^PV^ cells following CSA (two-way ANOVA, sex × treatment interaction, *F*_(1,39)_ = 12.05, *p* < 0.0013, main effect sex, *F*_(1,39)_ = 4.434, *p* = 0.0417, treatment main effect, *F*_(1,39)_ = 2.057, *p* = 0.160; Tukey’s *post hoc* test male naive vs male CSA, *p* = 0.006), and naive female rats exhibited a significantly lower frequency of sEPSCs compared with naive males (*p* = 0.005, Tukey’s). **p < 0.01, Tukey’s post-hoc test. Legend applies to ***C***, ***D***. ***C***, Mean (±95% CI) sEPSC amplitude in OFC^PV^ cells from male and female naive rats and those withdrawn from CSA. No changes in sEPSC amplitude were observed (two-way ANOVA, sex × treatment interaction, *F*_(1,38)_ = 0.064, *p* = 0.802). ***D***, Mean (±95% CI) effect of 5-HT (20 μm) on sEPSC frequency in OFC^PV^ cells from male and female naive Pvalb-iCre rats and those withdrawn from CSA. There was no effect of 5-HT on OFC^PV^ cells from naive or CSA-withdrawn rats (two-way ANOVA, sex × treatment interaction, *F*_(1,38)_ = 0.582, *p* = 0.450). ns, not significant.

The significantly higher frequency of sEPSCs in naive male rats compared with naive females noted in Figure 6 can also be seen in Figure 9*B*. After withdrawal from CSA, a significant decrease in sEPSC frequency was observed in OFC^PV^ neurons from male rats, but no changes in sEPSC frequency were seen in cells from female rats (Fig. 9*A*,*B*, two-way ANOVA, treatment × sex interaction, *F*_(1,39)_ = 12.05, *p* = 0.0013; sex main effect, *F*_(1,39)_ = 4.43, *p* = 0.0417; treatment main effect, *F*_(1,39)_ = 2.057, *p* = 0.1595; Tukey’s *post hoc* test, naive male vs CSA male, *p* = 0.0060; naive female vs CSA female, *p* = 0.9586). Withdrawal from CSA had no effect on sEPSC amplitudes (Fig. 9*C*, two-way ANOVA, treatment × sex interaction, *F*_(1,38)_ = 0.0639, *p* = 0.8018). As noted above, 5-HT had no effects on sEPSCs in OFC^PV^ neurons from naive animals (Fig. 6*D*,*E*). Similarly, after withdrawal from CSA, 5-HT failed to alter the amplitudes (means ± SEM, male CSA control = 19.35 ± 0.85 pA, CSA 5-HT = 18.81 ± 0.892 pA; female CSA control = 18.45 ± 0.99 pA, female CSA 5-HT = 19.27 ± 0.71 pA; data not shown) or frequencies of sEPSCs (Fig. 9*D*, sex × treatment interaction, two-way ANOVA *F*_(1,38)_ = 0.582, *p* = 0.450, main effect sex *F*_(1,38)_ = 0.355, *p* = 0.555, main effect of treatment *F*_(1,38)_ = 0.095, *p* = 0.760). Together, these data indicate that withdrawal from CSA is associated with a decrease in glutamatergic transmission onto OFC^PV^ interneurons in only male rats, and that 5-HT has no effect on glutamate release in these neurons in naive or CSA male or female Pvalb-iCre rats.

## Discussion

A growing body of evidence shows that 5-HT innervation of cortical areas is important for the expression of cognitive flexibility, as defined in tasks requiring a change in behavioral strategy to obtain reward. In marmosets, OFC 5-HT depletion impairs visual discrimination reversal (Clarke et al., 2004), and studies tapping similar cognitive domains in rodents show congruent results (Roberts, 2011; Izquierdo et al., 2012; West et al., 2013). More specifically, rodent studies show that 5-HT depletion produces deficits in behavioral tasks that require intact OFC 5-HT function to permit cognitive flexibility (Izquierdo et al., 2012; West et al., 2013; Roberts, 2011). Psychostimulants such as methamphetamine and cocaine also negatively affect OFC-dependent information processing, and in many cases, this is seen several weeks after cessation of drug exposure. Thus, long-lasting learning impairments in contingency reversal (Jentsch et al., 2002; Schoenbaum et al., 2004; Calu et al., 2007), reinforcer devaluation (Schoenbaum and Setlow, 2005), and in Pavlovian overexpectation tasks (Takahashi et al., 2013; Lucantonio et al., 2014) are reported. Moreover, the enduring nature of these deficits after withdrawal from self-administration of psychostimulants in animal models suggests that they may be involved in the dysregulated behavior that is observed in addiction to these drugs.

Links between cocaine and the 5-HT system have been documented at several levels of analysis. Cocaine is a potent inhibitor of the serotonin transporter (SERT; Torres et al., 2003), where it might act to alter 5-HT receptor function to indirectly alter OFC-dependent behavior. In support of this, the deleterious effects of CSA on OFC-dependent reversal learning are reduced in mice lacking the SERT (Nonkes et al., 2013), and some cocaine-induced behavioral changes are absent in mutant mice expressing SERT with reduced affinity for this drug (Simmler et., 2017). Based on these findings, it has been hypothesized that 5-HT signaling is involved in the contribution the OFC to cognitive flexibility, and that psychostimulant exposure may disrupt this 5-HT function in this capacity (Roberts, 2011; Wright et al., 2017; Hankosky et al., 2018). In this regard, our recent data also show profound changes to 5-HT signaling in OFC PyNs in rats withdrawn from CSA or yoked cocaine administration, and that this 5-HT hypofunction could be observed many weeks after withdrawal from cocaine (Wright et al., 2017). The changes included a decrease in 5-HT-induced inhibition of OFC PyNs via 5-HT_1A_ receptors, and a reduction in the ability of 5-HT_2A_ receptors to increase synaptic glutamate release. Although these data show 5-HT hypofunction in OFC PyNs following cocaine exposure, other potential sites of 5-HT interaction with the local OFC circuitry have not been studied. Thus, 5-HT_2_ receptors are also highly expressed on cortical GABAergic interneurons (Liu et al., 2007), and analogous fast-spiking parvalbumin cells in the medial PFC are depolarized by 5-HT_2A_ receptors (Weber and Andrade, 2010; Athilingam et al., 2017). To determine whether CSA alters 5-HT signaling in OFC^PV^ neurons, we developed a novel Pvalb-iCre transgenic rat line in which Cre recombinase expression is driven by the parvalbumin promoter. Cre-dependent expression of eYFP in the OFC of Pvalb-iCre rats was then used to study 5-HT signaling in OFC^PV^ cells in both naive rats and in those withdrawn from CSA. Additionally, as clinical studies show clear sex-dependent differences in vulnerability to CUD, with females more vulnerable to all phases of drug use, and similar results observed in animal models of addiction (Carroll and Anker, 2010), we powered our study to distinguishing potential differences between males and female Pvalb-iCre rats with regard to effects of CSA and altered 5-HT signaling.

Consistent with prior studies of PFC parvalbumin interneurons (Weber and Andrade, 2010; Athilingam et al., 2017), we found that 5-HT application caused depolarizing inward currents in the majority of OFC^PV^ cells in male and female Pvalb-iCre rats. However, this was blocked by the 5-HT_2_ receptor antagonist, ketanserin, in only females. The reason for this sex difference in ketanserin sensitivity in OFC^PV^ neurons is unknown. However, significantly greater binding of [^3^H]-ketanserin to 5-HT_2_Rs in all layers of the female rat hippocampus has been reported (Zhang et al., 1999), and this may be relevant in the female OFC. Alternatively, outward currents were observed in a minority of male OFC^PV^ neurons during 5-HT application in the present study (Fig. 5*C*) that were similar to outward currents mediated by 5HT_1A_ receptors in male rat PyNs (Wright et al., 2017). As this outward current may compete with the 5-HT-activated inward current, a partial blockade of inward current by ketanserin may be obscured. Another possibility may arise from the observations that ketanserin has higher affinity for 5-HT_2A_ versus 5-HT_2C_ receptors, and a low affinity for 5-HT_2B_ receptors (Barnes and Sharp, 1999). Therefore, sex-linked differences in the expression of these 5-HT_2_R subtypes could result in insensitivity to ketanserin in the male OFC^PV^ cells. Finally, differences in 5-HT_2A_ versus 5-HT_2C_ receptor expression on OFC^PV^ neurons between male and female rats have also been reported after methamphetamine self-administration (Hankosky et al., 2018), suggesting some sex-linked lability in the expression of 5-HT_2_R subtypes in these neurons. In addition to these differences in cellular distribution of 5-HT receptor subtypes, sexually dimorphic effects of 5-HT_2C_R and 5-HT_1A_R activation on CSA have been reported in rhesus monkeys (Collins and France, 2018). Therefore, data from the present and prior studies support the idea that 5-HT_2_R receptor subtype distribution differs between male and female mammals.

Sex-related differences were also noted in membrane and synaptic properties of OFC^PV^ neurons from naive rats and from those withdrawn from CSA. Thus, OFC^PV^ cells in naive female rats exhibited lower sEPSC baseline frequencies than males, suggesting that there is a lower probability of glutamate release onto these neurons in females. However, in contrast to the prominent increase in glutamate release by 5-HT reported in OFC PyNs (Wright et al., 2017), this was unaffected by 5-HT in OFC^PV^ cells in naive rats and those withdrawn from CSA. Despite this, OFC^PV^ cells in male Pvalb-iCre rats exhibited smaller holding currents, depolarized membrane potentials (Fig. 8*C*,*D*), and a reduction in sEPSCs following CSA (Fig. 9*B*). However, the inward currents caused by 5-HT were unchanged by CSA in OFC^PV^ cells from males and females (Fig. 8*G*,*H*). Although the effects of 5-HT on OFC^PV^ cells did not change after CSA withdrawal, the changes in OFC^PV^ neuron properties that were observed in males are likely to have profound effects on cortical circuit function and its participation in behavior. These changes in male rats could be compensatory and lead to a greater resistance to cocaine addiction compared with females (Carroll and Anker, 2010), or perhaps to an increased vulnerability to relapse to cocaine seeking triggered by conditioned cues under certain conditions, as reported in male rats (Fuchs et al., 2005). Collectively, our data show that male OFC^PV^ neurons are more sensitive to the effects of CSA itself, that 5-HT receptor subtypes may be differentially expressed on OFC^PV^ neurons of males and females, and, unlike that reported in OFC PyNs (Wright et al., 2017), the effects of 5-HT on OFC^PV^ cells are unaltered after CSA withdrawal. Therefore, these results suggest that withdrawal from cocaine exposure disrupts 5-HT signaling primarily at OFC PyNs, and that this may account for the dysregulated OFC-dependent behavioral and cognitive processes observed in CUD in humans and in animal models of cocaine addiction.

## Synthesis

Reviewing Editor: Mikko Airavaara, University of Helsinki Decisions are customarily a result of the Reviewing Editor and the peer reviewers coming together and discussing their recommendations until a consensus is reached. When revisions are invited, a fact-based synthesis statement explaining their decision and outlining what is needed to prepare a revision will be listed below. The following reviewer(s) agreed to reveal their identity: Stephanie Borgland, Donna Calu.

The reviewers have found your study interesting and potential for eNeuro. However, there are various points that need to be addressed. You have done a very good statistical table. Would it be possible to include this into the main text and not as supplement? Increasing the quality of statistical analysis is important for eNeuro. Also, please add reference to the methods part related to the table. Also, please present the statistical analysis/result for the reported slowing of initiation of inward currents in males. Mention how you did randomization, blinding, power analysis, and what was the exclusion criteria, and how many animals were excluded from the experiments. Please have figures appear in order of reference in the text (ie. Fig. 5D comes before Fig. 5C).

This study created a new rat transgenic line expressing cre recombinase in Pvalb neurons. They used this model to explore sex differences in the electrophysiological properties of orbitofrontal cortex Pvalb (OFCpv) neurons, their responsivity to serotonin, and possible changes associated with withdrawal from cocaine self administration. The authors go on to test the effects of serotonergic antagonists on the basal and synaptic properties of OFC PV neurons in naïve and cocaine withdrawn male and female rats. Using IHC and driving expression of Chr2 in the Cre expressing neurons, they demonstrate selective expression of Chr2 in PV neurons, which exhibit the expected physiological characteristics of PV neurons, demonstrated to be under tight control of optogenetic stimulation.

The most compelling aspect of this paper is the enormous sex difference on the difference in ability of ketanserin blocking 5HT effects on holding current only in female rats and the presence of outward currents induced by 5HT only in male rats. This suggest that there is a massive sex difference in 5HT receptor subtypes (or possibly coupling) on PV neurons between male and female rats. While this may be outside the scope of the current paper, this should be followed up. They also demonstrate that female OFCpv neurons have reduced sEPSC frequency, although it is unclear if this is due to a decreased release probability or a reduction in number of inputs.

Regardless, this reduced sEPSC frequency makes female OFCpv neurons less responsive to changes induced by cocaine self-administration withdrawal compared to male rats that have a decrease in sEPSC frequency due to cocaine self administration withdrawal. These effects appear to be independent of 5HT.

They also show that IPSCs are induced in nearby pyramidal cells, blocked by gabazine and likely monosynaptic as expected. Experiments uncover sex differences in spontaneous EPSC frequency for OFC PV neurons as well as sex-specific effects of 1) 5HT2A/2C receptor antagonist on inward currents in OFC PV neurons and 2) cocaine-withdrawal on basal properties of OFC PV neurons.

Specific concerns:

1. Development of new new transgenic rat line to target PV neurons is appreciated. Since this paper will likely be referenced if the rats are used by others, it would be helpful to have a more fulsome characterization and quantification of the efficiency and accuracy of expression. For example, they should quantify their immunohistochemistry experiments and determine 1) how many PV+ neurons express icre? How many non-PV+ neurons express cre (ie is there any leaky expression?) Is there variation in expression in OFC subregions? Is it also expressed in PV neurons outside the OFC?

2. The impact of these findings could be strengthened by interpreting the results (sex differences, relative absence of cocaine effects) in the context of what is known or was expected/hypothesized at the outset of the experiments. Discussion of methodological limitations should address a variety of factors, including food restriction in CSA rats, operant experience, cocaine self-administration, and withdrawal, any of which could account for the observed effects and/or sex-differences. The absence of yoked and/or operant controls in the CSA experiments, while arguably outside the scope of the current study, are limitations and should be discussed. Important to this discussion, details about the cocaine self-administration behaviors must be added to the results section. A timeline of behaviors/tissue harvest, as well as basic operant data for males and females would assist reader in interpreting results from this part of the study.

3. The finding of 5-HT receptor function differs between sexes in interesting, but is not explored further experimentally, and is unaccounted for in the discussion. Are there differences in 5-HT2A/2C/1A receptor expression in PV+ neurons between male and females? Is it possible differential transgene expression in subsets of PV cells with varying receptor expression could account for differences between the sexes? Is the baseline difference in EPSC frequency related to the sex difference in 5-HT blockade by ketanserin? Importantly - could the frequency decrease of EPSCs in males following cocaine use be associated with a change in ketanserin sensitivity? Are changes in 5-HT receptor function known to result from cocaine exposure/self-administration? Is there any published single cell RNA sequencing data available on 5HT transcript levels in PV neurons that could be further analysed or discussed?

4. In figure 5, they have presented the baseline effects of 5HT suppression of holding current in both C and D in addition to A. This gives the impression they have done one experiment to test for sex difference and then repeated the experiment to test for inhibition by ketanserin. However, this was one experiment where they have reused the controls. The ketanserin data should be presented together with the baseline responses (ie remove figure 5A) and then add a bargraph with maximal effects of 5HT +/- Ketanserin in male and female rats. They can test for a sex x drug interaction and main effects of sex or drug on 5HT inhibition of holiding current using a 2 way ANOVA.

5. Figure 6C and D should be analyzed with a 2 way ANOVA to determine if there is sex x drug interaction on sEPSCs or main effects of sex or serotonin. In addition to male Control + 5HT, show female control + 5HT traces. The difference in male vs female sEPSC frequency would likely be observed in the control traces.

6. Figures 8A, B should be analyzed with a 2 way ANOVA as they are exploring interactions between sex and CSA on RMP or holding current. Please add example traces for firing frequency.

7. Figures 9B, C, D should be analyzed with a 2 way ANOVA as they are exploring interactions between sex and CSA on sEPSCs

8. For the 2-Way ANOVA listed in line 311 (eYFP and current injection), please list the p value and the main effects.

8. For the rats with cocaine self administration, could they add a figure showing the cocaine intake over the 12 daily sessions?

10. Methods:

How long were the rats withdrawn/abstinent before electrophysiology experiments? This information should be listed in the methods.

10. Indicate where they purchased their ChR2 virus vector (line 163) and where they obtain the 5HT (line 269).

11. While external and internal solutions were listed in the electrophysiological methodology, they did not indicate which drugs they used in the bath to isolate GABAergic or glutamatergic inputs for oIPSCs and sEPSCs. Please list the criteria used to accept sEPSC wave forms (ie rise time, amplitude, decay time, etc).

12. Methods lines 178-179: “They were maintained in a temperature- and humidity-controlled environment under a reverse 12 h light/dark cycle with food and water available ad libitum”. Mention when the lights went on/off. Were the experiments always carried out at the same time of the day?

## Author Response

Synthesis Statement for Author (Required):

The reviewers have found your study interesting and potential for eNeuro. However, there are various points that need to be addressed.

1. You have done a very good statistical table. Would it be possible to include this into the main text and not as supplement? Increasing the quality of statistical analysis is important for eNeuro. Also, please add reference to the methods part related to the table.

This is now included as Table 2 at the end of the Results section, and it is mentioned in the Methods.

2. Also, please present the statistical analysis/result for the reported slowing of initiation of inward currents in males. Mention how you did randomization, blinding, power analysis, and what was the exclusion criteria, and how many animals were excluded from the experiments. Done Please have figures appear in order of reference in the text (ie. Fig. 5D comes before Fig. 5C).

This statistical information is now included in Methods. The figures are now in the proper order.

This study created a new rat transgenic line expressing cre recombinase in Pvalb neurons. They used this model to explore sex differences in the electrophysiological properties of orbitofrontal cortex Pvalb (OFCpv) neurons, their responsivity to serotonin, and possible changes associated with withdrawal from cocaine self administration. The authors go on to test the effects of serotonergic antagonists on the basal and synaptic properties of OFC PV neurons in naïve and cocaine withdrawn male and female rats. Using IHC and driving expression of Chr2 in the Cre expressing neurons, they demonstrate selective expression of Chr2 in PV neurons, which exhibit the expected physiological characteristics of PV neurons, demonstrated to be under tight control of optogenetic stimulation.

The most compelling aspect of this paper is the enormous sex difference on the difference in ability of ketanserin blocking 5HT effects on holding current only in female rats and the presence of outward currents induced by 5HT only in male rats. This suggest that there is a massive sex difference in 5HT receptor subtypes (or possibly coupling) on PV neurons between male and female rats. While this may be outside the scope of the current paper, this should be followed up. They also demonstrate that female OFCpv neurons have reduced sEPSC frequency, although it is unclear if this is due to a decreased release probability or a reduction in number of inputs.

Regardless, this reduced sEPSC frequency makes female OFCpv neurons less responsive to changes induced by cocaine self-administration withdrawal compared to male rats that have a decrease in sEPSC frequency due to cocaine self administration withdrawal. These effects appear to be independent of 5HT.

They also show that IPSCs are induced in nearby pyramidal cells, blocked by gabazine and likely monosynaptic as expected. Experiments uncover sex differences in spontaneous EPSC frequency for OFC PV neurons as well as sex-specific effects of 1) 5HT2A/2C receptor antagonist on inward currents in OFC PV neurons and 2) cocaine-withdrawal on basal properties of OFC PV neurons.

Specific concerns:

1. Development of new new transgenic rat line to target PV neurons is appreciated. Since this paper will likely be referenced if the rats are used by others, it would be helpful to have a more fulsome characterization and quantification of the efficiency and accuracy of expression. For example, they should quantify their immunohistochemistry experiments and determine 1) how many PV+ neurons express icre? How many non-PV+ neurons express cre (ie is there any leaky expression?) Is there variation in expression in OFC subregions? Is it also expressed in PV neurons outside the OFC?

To address these points, we conducted additional Pvalb-iCre rat characterization experiments. To do this, we injected the AAV Nuc flox (mCherry)-eGFP construct into the OFC of 3 male and 3 female Pvalb-iCre rats and then examined co-expression of parvalbumin with green and red fluorescence signal four weeks later.

We observed that the percentage of red (mCherry, i.e. un-recombined by Cre) cells, that colocalized with parvalbumin antibody was less than 5%. The percentage of eGFP cells (i.e. recombined by Cre) coexpressing parvalbumin varied from 20% to 80% across animals. Quantification revealed the percentage of eGFP-positive cells coexpressing parvalbumin antibody was variable but much higher than cells coexpressing mCherry and parvalbumin (Fig. 1D). The lack of parvalbumin immunoreactivity in some eGFP expressing cells may be due to variability in parvalbumin expression, and therefore Cre, relative to when the injection occurred. The ability to colocalize parvalbumin antibody signal with nuclear fluorescent signal may also be a limiting factor. However, importantly, cells that showed no signs of recombination (mCherry positive cells) consistently showed minimal overlap with parvalbumin expression (Fig. 1B, 1D). Moreover, neurons located with eYFP fluorescence following injection with Cre-dependent AAV5-EF1α-DIO-hChR2(H134R)-EYFP into the OFC of Pvalb-iCre rats uniformly exhibited distinctive electrophysiological properties that are characteristic of fast-spiking parvalbumin neurons identified throughout the rodent CNS (e.g. DeFelipe et. al. New insights into the classification and nomenclature of cortical GABAergic interneurons. Nature reviews Neuroscience. 2013;14(3):202-16. pmid:23385869; Hu et. al. Interneurons. Fast-spiking, parvalbumin(+) GABAergic interneurons: from cellular design to microcircuit function. Science. 2014;345(6196):1255263. Epub 2014/08/02. pmid:25082707).

Is there variation in expression in OFC subregions?

Our electrophysiological recordings were confined to layer 5 of the OFC and we did not characterize parvalbumin-driven Cre expression outside of this layer. However, parvalbumin neurons have been described in all layers of cerebral cortex in rodents and primates where they are typically described as basket cells or chandelier inhibitory neurons (Kawaguchi and Kubota. GABAergic cell subtypes and their synaptic connections in rat frontal cortex. Cereb Cortex. 1997 7:476-86. doi: 10.1093/cercor/7.6.476. PMID: 9276173; DeFelipe et. Al., Visualization of chandelier cell axons by parvalbumin immunoreactivity in monkey cerebral cortex. Proc Natl Acad Sci U S A. 1989, 86:2093-7. doi: 10.1073/pnas.86.6.2093. PMID: 2648389; PMCID: PMC286854).

Is it also expressed in PV neurons outside the OFC?

We have performed qualitative analyses for Cre expression outside of the OFC in the Pvalb-iCre rat and found that both somatosensory cortex and midbrain (non-dopaminergic neurons) show subsets of neurons that promote Cre-recombination. This has now been noted in the manuscript. In addition, Dr. Gavan McNally's lab (University of New South Wales) has characterized Pvalb-iCre rats for studies in the basolateral amygdala. In these studies, they have measured colocalization of eYFP and Parvalbumin in basolateral amygdala following injection with Cre-dependent AAV- halorhodpsin-eYFP (confidential unpublished figure below) or AAV-eYFP. They found that 98% of eYFP positive cells colocalize with PV. We have now noted these unpublished observations in the results. We have also noted in the results the importance for anyone using this rat (or any transgenic rat) to verify Cre-dependent transgene expression in the region of interest.

2. The impact of these findings could be strengthened by interpreting the results (sex differences, relative absence of cocaine effects) in the context of what is known or was expected/hypothesized at the outset of the experiments.

Discussion of methodological limitations should address a variety of factors, including food restriction in CSA rats, operant experience, cocaine self-administration, and withdrawal, any of which could account for the observed effects and/or sex-differences. The absence of yoked and/or operant controls in the CSA experiments, while arguably outside the scope of the current study, are limitations and should be discussed.

Our null hypothesis at the outset of these experiments was that no differences between males and females would be observed, simply because there is not much literature on the cellular effects of 5-HT in cortical cells in cocaine addiction models. Therefore, at the outset we had no reason to suspect such differences would be present. Regarding a more general hypothesis for these experiments, we predicted, based on published data with OFC PyNs, that the 5-HT responsiveness of OFCPV neurons would be decreased or eliminated after withdrawal from CSA. Obviously, and somewhat surprisingly, this hypothesis was not supported. We have now included the following sentence stating this hypothesis near the end of the Introduction “ As OFCPV neurons also express 5-HT receptors (Santana et al., 2004; Athilingam et al., 2017), and promote cortical synchrony (Puig et al., 2010), we hypothesize that withdrawal from CSA will also alter 5-HT responses in these cells.”

Some of these issues regarding limitations of cocaine control groups and behavioral history can be explained in the context of a prior publication in which CSA rats were compared with yoked controls and naïve rats (Wright et al., 2017). In this study, yoked control animal PyNs showed changes in 5-HT sensitivity that were comparable to those in the CSA group, and therefore likely a consequence of exposure to cocaine per se. Therefore, in the present study a yoked control group was not included, because we would hypothesize that a yoked ocaine control group would have yielded similar results to the CSA group. Moreover, addition of a yoked control group would have required many additional transgenic rats that at present represent a limited resource.

Additionally, as the most interesting findings from our study indicate difference between male and female transgenic CSA animals, or differences between male or female CSA rats and their naïve transgenic counterparts, we feel that most potential mitigating factors, such as food restriction, operant experience etc., have strong internal controls. For instance, the observation that ketanserin sensitivity differs between male and female OFCPV neurons in naïve animals requires no additional controls for these other factors. Similarly, the observation that CSA withdrawal results in decreased RMP, increased Ihold, and a decrease in sEPSCs in male, and not female OFCPV neurons, would seem to suggest that procedural differences between naïve and CSA animals were not involved in these effects. Therefore, in a sense, the absence of changes in the female CSA animals serves as a control for the changes relative to naïve rats observed in the CSA males.

Important to this discussion, details about the cocaine self-administration behaviors must be added to the results section. A timeline of behaviors/tissue harvest, as well as basic operant data for males and females would assist reader in interpreting results from this part of the study.

We have added data regarding self-administration and the total amount of cocaine intake for both male and female rats to the Results, and to Fig. 8A and 8B. We have also added extensive additional data beginning on line 430 of the Results indicating the number of days of withdrawal from CSA before beginning the electrophysiology experiments.

3. The finding of 5-HT receptor function differs between sexes in interesting, but is not explored further experimentally, and is unaccounted for in the discussion.

We have considered these results in the Discussion and in the context of recent findings regarding differences in 5-HT receptor expression and function in male and female animals. However, it should be noted that whereas there is some literature on sexually dimorphic 5-HT function in males and females, the observations are not specific to 5HT-2 receptors (except where noted in the discussion of our paper) and therefore, we feel are too broad to be considered extensively in the present context.

Are there differences in 5-HT2A/2C/1A receptor expression in PV+ neurons between male and females? Is it possible differential transgene expression in subsets of PV cells with varying receptor expression could account for differences between the sexes?

It is possible that there are differences in the expression of these receptor subtypes on parvalbumin neurons in the OFC and we consider this in the Discussion. Unfortunately, there is a limited literature in this regard. However, one study shows in the hippocampus that 3H-ketanserin binding is lower in male vs female rats (Zhang et al., 1999 cited in the manuscript), and there are behavioral pharmacology studies supporting differences without regional specificity. This is clearly an issue that requires closer scrutiny. As to the possibility of differential transgene expression in subsets of cells, of course this is always a concern (or fear!) when working with transgenic organisms. However, at this point we do not see differences at our current level of analysis (see additional data in response to point #1 above).

Is the baseline difference in EPSC frequency related to the sex difference in 5-HT blockade by ketanserin? Importantly - could the frequency decrease of EPSCs in males following cocaine use be associated with a change in ketanserin sensitivity?

As the magnitude of the inward currents caused by 5-HT did not differ after CSA withdrawal in either females or males, we have no reason to suspect that ketanserin sensitivity would be differentially altered by CSA. Moreover, as the sEPSC decrease in male OFCPV neurons after withdrawal from CSA was noted in the absence of 5-HT, and unchanged by application of the monoamine, it does not seem that there is a relationship between 5-HTRs and this effect.

Are changes in 5-HT receptor function known to result from cocaine exposure/self-administration? Is there any published single cell RNA sequencing data available on 5HT transcript levels in PV neurons that could be further analysed or discussed?

A study published in 2017 showed that withdrawal from CSA or yoked cocaine administration was associated with an increase in 5-HT2A mRNA in several frontocortical brain areas, including OFC, and that 5-HT1A transcripts were unaffected (Wright et al., 2017). However, this was done with in situ hybridization and not at the level of single cells. Therefore, cell type specific changes were not reported. To our knowledge, other than studies establishing the presence of 5-HTR transcripts in PFC parvalbumin neurons, no effects of cocaine on this expression have been reported.

4. In figure 5, they have presented the baseline effects of 5HT suppression of holding current in both C and D in addition to A. This gives the impression they have done one experiment to test for sex difference and then repeated the experiment to test for inhibition by ketanserin. However, this was one experiment where they have reused the controls. The ketanserin data should be presented together with the baseline responses (ie remove figure 5A) and then add a bargraph with maximal effects of 5HT +/- Ketanserin in male and female rats. They can test for a sex x drug interaction and main effects of sex or drug on 5HT inhibition of holiding current using a 2 way ANOVA.

This 2-way ANOVA has been performed and is now reported in the manuscript. In addition, the changes to Fig. 5 that were suggested by the reviewer have been made.

5. Figure 6C and D should be analyzed with a 2 way ANOVA to determine if there is sex x drug interaction on sEPSCs or main effects of sex or serotonin. In addition to male Control + 5HT, show female control + 5HT traces. The difference in male vs female sEPSC frequency would likely be observed in the control traces.

The 2-way ANOVA has been performed and is now reported in the manuscript.

This difference between males and females in the control condition is shown in Fig. 6A.

6. Figures 8A, B should be analyzed with a 2 way ANOVA as they are exploring interactions between sex and CSA on RMP or holding current. Please add example traces for firing frequency.

The 2-way ANOVA has been performed and is now reported in the manuscript. Example traces of firing frequency have been added to Fig. 8E and 8F.

7. Figures 9B, C, D should be analyzed with a 2 way ANOVA as they are exploring interactions between sex and CSA on sEPSCs.

The 2-way ANOVA has been performed and is now reported in the manuscript.

8. For the 2-Way ANOVA listed in line 311 (eYFP and current injection), please list the p value and the main effects.

This has been added to the manuscript.

8. For the rats with cocaine self administration, could they add a figure showing the cocaine intake over the 12 daily sessions?

Information regarding daily cocaine self-administration in males and females has been added to Fig. 8A. In addition, the total cocaine intake for male and female rats is now reported in Fig. 8B. There were no differences.

10. Methods:

How long were the rats withdrawn/abstinent before electrophysiology experiments? This information should be listed in the methods.

Rats were withdrawn from CSA for 55.7 {plus minus} 5.7 days (mean {plus minus} s.e.m). Male and female rats were withdrawn from CSA for a similar number of days (45.8 {plus minus} 4.6, and 64.6 {plus minus} 9.4 days, respectively, t17 = 1.733, p = 0.101, unpaired t-test). This information is now included in the Results.

10. Indicate where they purchased their ChR2 virus vector (line 163) and where they obtain the 5HT (line 269).

This information has been added to Methods.

11. While external and internal solutions were listed in the electrophysiological methodology, they did not indicate which drugs they used in the bath to isolate GABAergic or glutamatergic inputs for oIPSCs and sEPSCs. Please list the criteria used to accept sEPSC wave forms (ie rise time, amplitude, decay time, etc).

This information has been added to Methods

12. Methods lines 178-179: “They were maintained in a temperature- and humidity-controlled environment under a reverse 12 h light/dark cycle with food and water available ad libitum”. Mention when the lights went on/off. Were the experiments always carried out at the same time of the day?

The lights were turned off at 9 am and turned on at 9 pm. All behavioral experiments were run during the dark phase of the light cycle and begun each day between 10-12 am. This information has been added to Methods.
